# Visualization of individual cell division history in complex tissues using iCOUNT

**DOI:** 10.1016/j.stem.2021.08.012

**Published:** 2021-11-04

**Authors:** Annina Denoth-Lippuner, Baptiste N. Jaeger, Tong Liang, Lars N. Royall, Stefanie E. Chie, Kilian Buthey, Diana Machado, Vladislav I. Korobeynyk, Merit Kruse, Clara M. Munz, Alexander Gerbaulet, Benjamin D. Simons, Sebastian Jessberger

**Affiliations:** 1 Laboratory of Neural Plasticity, Faculties of Medicine and Science, Brain Research Institute, University of Zurich, 8057 Zurich, Switzerland; 2 Institute for Immunology, Faculty of Medicine Carl Gustav Carus, Technische Universität Dresden, 01307 Dresden, Germany; 3 Wellcome Trust-Medical Research Council Stem Cell Institute, University of Cambridge, Cambridge CB2 1QR, UK; 4 The Wellcome Trust/Cancer Research UK Gurdon Institute, University of Cambridge, Cambridge CB2 1QN, UK; 5 Department of Applied Mathematics and Theoretical Physics, Centre for Mathematical Sciences, University of Cambridge, Wilberforce Road, Cambridge CB3 0WA, UK

**Keywords:** Cell division history, stem cell proliferation, neurogenesis, single cell RNA sequencing, imaging, recombination, transgenesis, human brain organoid

## Abstract

The division potential of individual stem cells and the molecular consequences of successive rounds of proliferation remain largely unknown. Here, we developed an inducible cell division counter (iCOUNT) that reports cell division events in human and mouse tissues *in vitro* and *in vivo*. Analyzing cell division histories of neural stem/progenitor cells (NSPCs) in the developing and adult brain, we show that iCOUNT can provide novel insights into stem cell behavior. Further, we use single-cell RNA sequencing (scRNA-seq) of iCOUNT-labeled NSPCs and their progenies from the developing mouse cortex and forebrain-regionalized human organoids to identify functionally relevant molecular pathways that are commonly regulated between mouse and human cells, depending on individual cell division histories. Thus, we developed a tool to characterize the molecular consequences of repeated cell divisions of stem cells that allows an analysis of the cellular principles underlying tissue formation, homeostasis, and repair.

## Highlights

• iCOUNT reports previous cell divisions in mouse and human cells *in vitro*
• iCOUNT detects cell division biographies in complex mouse tissues *in vivo*
• iCOUNT allows for the analysis of human progenitors in forebrain organoids
• scRNA-seq of iCOUNT cells identifies molecular consequences of previous cell divisions

## Graphical abstract

**Figure:**
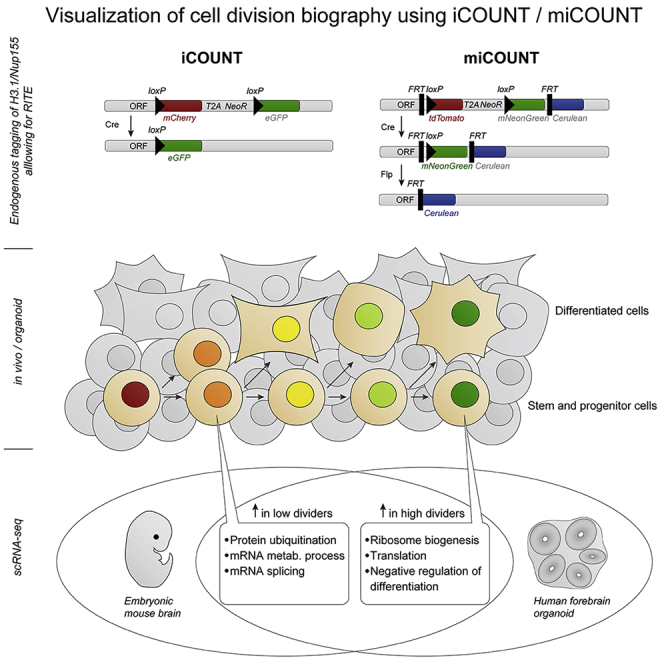

## Teaser

In this study, Denoth-Lippuner and colleagues developed an inducible cell division counter (iCOUNT) that reports the number of previous divisions of stem cells *in vitro* and *in vivo*. Using the iCOUNT, they identify molecular changes occurring with increased cell division history in the mouse developing brain and in human brain organoids.

## Introduction

Somatic stem cell proliferation does not end with embryogenesis, as many tissues such as skin, intestines, the blood system, and the central nervous system continue to rely on somatic stem cells for tissue homeostasis and repair (Barker, 2014; Bianconi et al., 2013; Brack and Rando, 2012; Donati and Watt, 2015; Gage, 2019; Morrison and Spradling, 2008). Despite increasing knowledge about lineage relationships of somatic stem cells based on advances in cellular barcoding and imaging (Fuentealba et al., 2015; Kalhor et al., 2018; Mayer et al., 2015; McKenna et al., 2016; Park et al., 2016), the functional and molecular consequences of previous cellular experiences, such as cell division events, remain largely unknown. Clearly, biographical events may be important to govern the behavior of individual cells and their response to external stimuli (Royall and Jessberger, 2021). Therefore, a number of tools have been developed recently with the aim of recording single-cell biographies based on a variety of potential experiences, such as the previous activity of multiple signaling pathways or even complete transcriptional profiles (Farzadfard and Lu, 2018; Frieda et al., 2017; Schmidt et al., 2018). These approaches have been successful in the context of cultured cells and bacteria using genetic approaches that allow turning back time and looking into the past of individual cells (Frieda et al., 2017; Schmidt et al., 2018; Tang and Liu, 2018). However, a transfer of those or related technologies into more complex tissues (e.g., organoids) or even to the *in vivo* situation in mammals is missing. Previous rounds of cell divisions represent a key cellular experience of individual stem cells during organ development, tissue homeostasis, and stem-cell-based cellular repair. However, how single cells respond to physiological or disease-associated stimuli, and how this response depends on an individual cell’s division history, remains largely unknown. Previously developed techniques to assess the number of cell divisions such as the dilution of fluorescent dyes (e.g., carboxyfluorescein-succinimidyl-ester [CFSE]) or overexpressed histone H2B fused to GFP (H2B-GFP) are valuable tools to identify label-retaining cells, label cohorts of cells to be tracked, or assess the number of previous cell divisions *in vitro* or in transplanted cells (Lyons and Parish, 1994; Tumbar et al., 2004). However, CFSE requires stem cell isolation and re-implantation into the niche (e.g., of hematopoietic stem cells), which artificially activates quiescent cells or is only applicable within a few specific stem cell niches (e.g., developing neocortex using FlashTag) (Cilloni et al., 2020; Takizawa et al., 2011; Telley et al., 2016). Overexpression of H2B-GFP *in vivo* was reported to be leaky in quiescent stem cells, to be diluted independent of cell division, to be loaded non-homogenously allowing analyses only on a population level, to alter cell proliferation, and to affect chromatin organization and thereby gene transcription and the animal’s behavior (Bernitz et al., 2016; Challen and Goodell, 2008; Ito et al., 2014; Meeks-Wagner and Hartwell, 1986; Morcos et al., 2020; Toyama et al., 2019). These deficiencies limit the potential of the H2B-GFP system to operate reliably as a quantitative assay to record the history of individual cell activity. To overcome these limitations, here we developed a genetic tool, the iCOUNT, that allows a precise measure of previous cell division events in complex mouse tissues and human organoids on a single-cell level.

## Results

### iCOUNT reports cell division events

**Figure 1:**
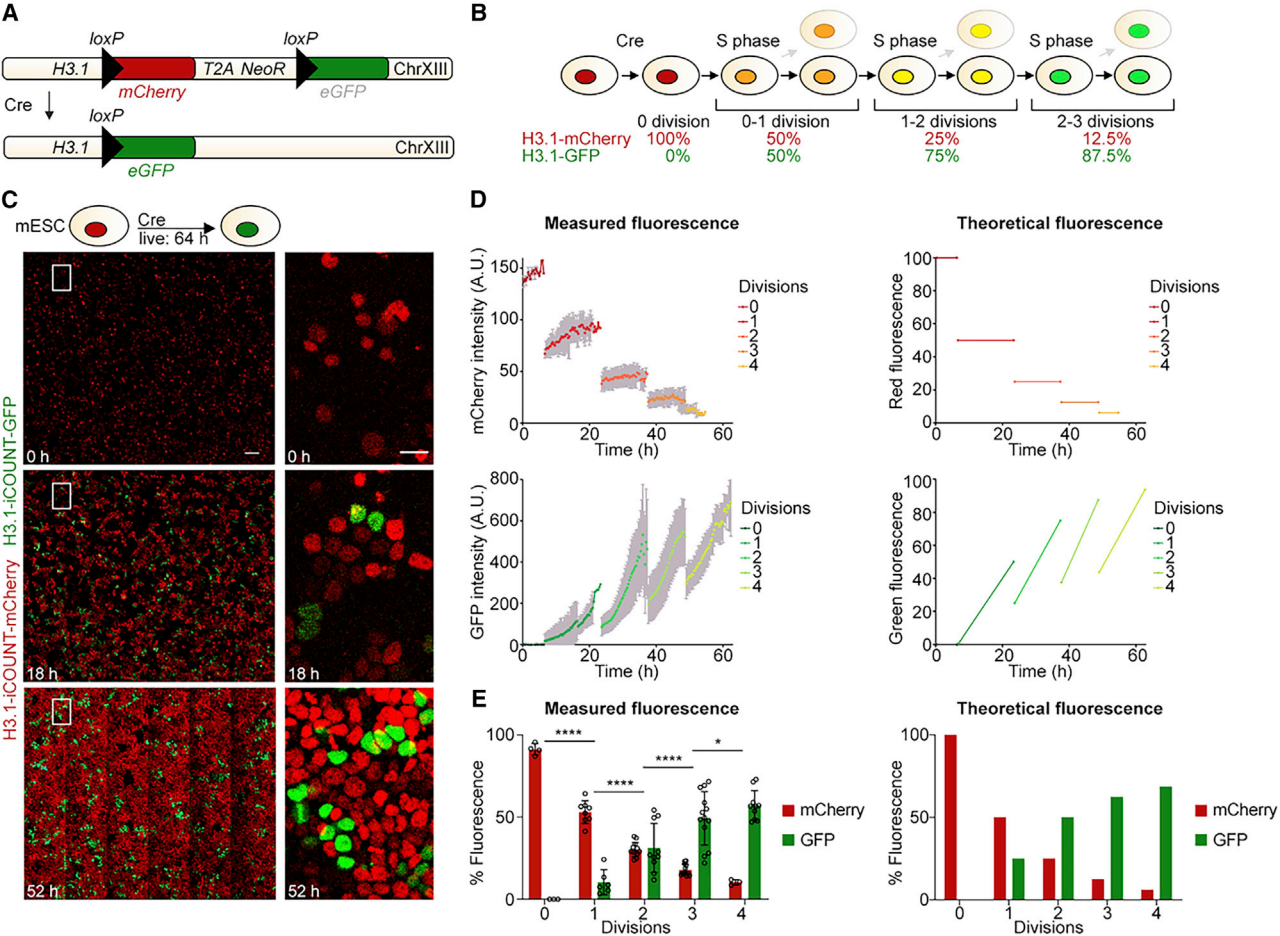
iCOUNT reports cell division events (A) iCOUNT genetic knockin design. (B) Theoretical change in fluorescent ratios with subsequent divisions in iCOUNT-targeted cells. (C) Selected time points of live imaging of mouse ESCs for 64 hours. Right panels show high magnifications of boxed areas. (D) Measured (left) and theoretical (right) changes in fluorescence intensities for red (mCherry) and green (GFP) histones of single cells (mean ± SD). (E) Quantification of measured (left) and theoretical (right) changes in percentage of fluorescence for red and green histones of iCOUNT-targeted cells (mean ± SD; open circles depict values of single cells). n = 4 starting cells and n ≥ 4 daughter cells for each further cell division (D and E). ^∗^p < 0.05; ^∗∗∗∗^p < 0.0001. Images were stitched. Scale bars represent 100 μm (C, left panels) and 20 μm (C, right panels). See also Figure S1 and Video S1.

To identify the cell division history of individual cells in complex tissues, we generated an inducible cell division counter (iCOUNT). The iCOUNT approach uses recombination-induced tag exchange (RITE) of endogenously tagged cell-cycle-dependent proteins such as histone variant H3.1 and NUP155, allowing for a Cre-dependent switch from a red to a green fluorescent-tagged protein, as shown for a switch from H3.1-mCherry to H3.1-GFP (Figure 1A) (Toyama et al., 2013; Verzijlbergen et al., 2010). We hypothesized that, after the addition of Cre recombinase, every subsequent cell division would reduce the amount of pre-existing red histones by one half and refill the pool of histones with newly synthesized green histones, thus allowing the number of previous cell divisions to be inferred from the changes in red/green ratios (Figure 1B).

We first tested the iCOUNT system in mouse embryonic stem cells (mESCs). mESCs with H3.1-tagged iCOUNT expressed red histones, and 48 hours after co-transfection of a Cre and Cre-reporter plasmid (lox-STOP-lox-mKO2), 81% of Cre-reporter expressing cells switched to green histones (Figure S1A). Fluorescent time-lapse imaging of iCOUNT expressing mESCs over a period of 64 hours (Figure 1C; Video S1) revealed that the red fluorescence dropped by half on every cell division and was refilled by green fluorescence (Figures 1D and 1E, left panels, and S1F), confirming the theoretically expected values (Figures 1D and 1E, right panels). Tagged histones were symmetrically segregated between daughter cells (Figure S1B), and tagging H3.1 did not affect proliferation (as measured by the incorporation of 5-Ethynyl-2′-deoxyuridine [EdU]), apoptosis (as measured by cleaved Caspase-3 labeled cells), genomic integrity (as measured by phosphorylated H2AX signal), or gene expression of ESCs (Figures S1C–S1E). Comparing the directly measured division history of individual cells with the analysis of the percentage of green histones at each time point showed that the iCOUNT system correctly predicted 95.5% of all division events (Figure S1G). Using fluorescence-activated cell sorting (FACS), we analyzed long-term dynamics (6 days post-Cre) of the color exchange at the population level and found a shift from red to green fluorescence in cells transfected with Cre-recombinase-expressing plasmids (Figures S1H–S1I).

Video S1. mESCs expressing H3.1-iCOUNT imaged for 64 h starting 6 h post-transfection with Cre recombinase, related to Figure 1 Movie shows the gradual change from red (mCherry) to green (GFP)-tagged histones in cells that underwent Cre-dependent recombination. Duration of the movie: 64 h. Tiles were stitched. Scale bar represents 100 μm.

**Figure 2:**
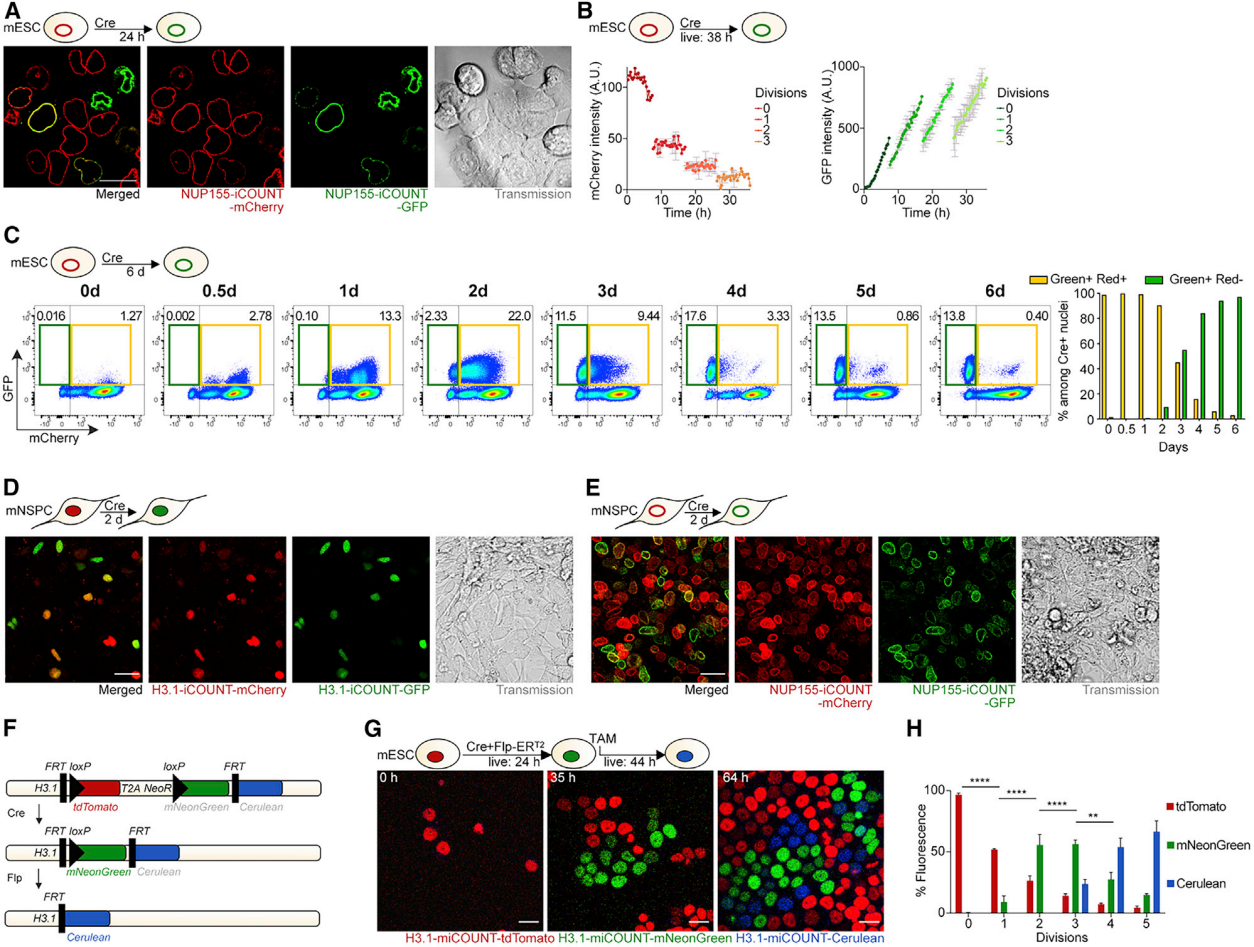
iCOUNT and miCOUNT signals in diverse mouse cell types (A) Expression of mCherry- (red) and GFP-tagged (green) nuclear pore proteins NUP155 in mouse ESCs 24 hours after Cre-mediated recombination. (B) Measured changes in fluorescence intensities for red (mCherry) and green (GFP) NUP155 of single cells (mean ± SD). (C) FACS analyses of NUP155 show relative shift from red/green to green fluorescent intensities in mouse ESCs in the course of 6 days after Cre-mediated recombination. Quantification of measured changes (boxed areas: orange depicts mCherry+ GFP+ cells and green depicts mCherry- GFP+ cells) in fluorescence intensities for red and green NUP155-iCOUNT-expressing cells. (D) Expression of mCherry- (red) and GFP-tagged (green) histones in mouse NSPCs 2 days after Cre-mediated recombination. Left panel shows merged signal. (E) Expression of mCherry- (red) and GFP-tagged (green) NUP155 in mouse NSPCs 2 days after Cre-mediated recombination. Left panel shows merged signal. (F) miCOUNT genetic knockin design. (G) Selected time points of live imaging of miCOUNT-targeted cells for 68 hours. (H) Quantification of fluorescence intensities for red (tdTomato), green (mNeonGreen), and blue (Cerulean) histones of miCOUNT-targeted mouse ESCs grouped by cell divisions (mean ± SD). Note the initial increase in newly synthesized green histones in miCOUNT-targeted cells after Cre-mediated recombination that is then replaced by newly synthesized blue histones after induction of Flp. n = 1 starting cell and n ≥ 2 daughter cells for each further cell division (B); n ≥ 3 cells (H). ^∗∗^p < 0.01; ^∗∗∗∗^p < 0.0001. Images were stitched. Scale bars represent 20 μm. See also Figure S2 and Video S2.

To test the versatility of RITE-based cell division counting, we tagged NUP155, a stable core component of the nuclear pore complex, with the iCOUNT cassette (Toyama et al., 2013). NUP155-iCOUNT showed a similar color exchange as H3.1 after transfection with Cre expressing plasmids, as measured by time-lapse imaging and FACS analysis in mESCs (Figures 2A–2C). To test the ability of iCOUNT to report cell divisions in other cell types, we generated H3.1-iCOUNT and NUP155-iCOUNT mouse hippocampal neural stem/progenitor cells (mNSPCs) that again showed robust color exchange after electroporation of plasmids expressing Cre recombinase (Figures 2D and 2E). An important feature of a cell division counter is its stability in non-dividing cells. To compare iCOUNT to previously used methods, the fluorescent signals of iCOUNT, CFSE, and overexpressed H2B-GFP were measured in non-dividing cells. First, mNSPCs were differentiated into neurons (by withdrawing growth factors [GFs]) or quiescence was induced by adding BMP4 (Mira et al., 2010). Then, fluorescence was measured daily by FACS. iCOUNT NSPCs showed a color exchange after electroporation with Cre expressing plasmids while proliferating, which ceased when cells became quiescent or differentiated into neurons (Figures S2A–S2D). In contrast, fluorescence dropped continuously in CFSE-labeled cells or cells loaded with H2B-GFP, even when cells were non-dividing (FACS analysis starting on day 3 and adding EdU overnight to exclude the small proportion of still dividing cells; Figure S2E). In addition, quiescent NSPCs were imaged over 5 days. Whereas quiescent cells loaded with H2B-GFP continuously lost fluorescence, red/green fluorescent ratios remained stable in Cre-treated iCOUNT cells (Figures S2F and S2G).

Finally, we designed a multiple iCOUNT (miCOUNT) that allows a first, Cre-dependent color switch (red to green), followed by a second, Flipase (Flp)-dependent color switch (green to blue; Figure 2F). mESCs expressing H3.1-miCOUNT were transduced with Cre and tamoxifen (TAM)-inducible Flp, and time-lapse imaging revealed first a switch from red (tdTomato) to green (mNeonGreen) fluorescence, followed by a color exchange from green to blue (Cerulean) fluorescence after the addition of TAM (24 hours after the start of imaging; Figures 2G and 2H; Video S2). Taken together, we show that the iCOUNT system, using different tagged proteins in different cell types, reliably reports the number of previous cell division events *in vitro*.

Video S2. mESCs expressing H.3-miCOUNT were co-transfected with Cre and TAM-inducible Flp 6 h prior to imaging, related to Figure 2 24 h after imaging started, TAM was added and live imaging continued for additional 44 h. The movie shows first a gradual change from red (tdTomato) to green (mNeonGreen) histones followed by a change from green to blue (Cerulean) histones. Duration of the movie: 68 h. Tiles were stitched. Scale bar represents 20 μm.

### *In vivo* visualization of individual cell division histories using iCOUNT

**Figure 3:**
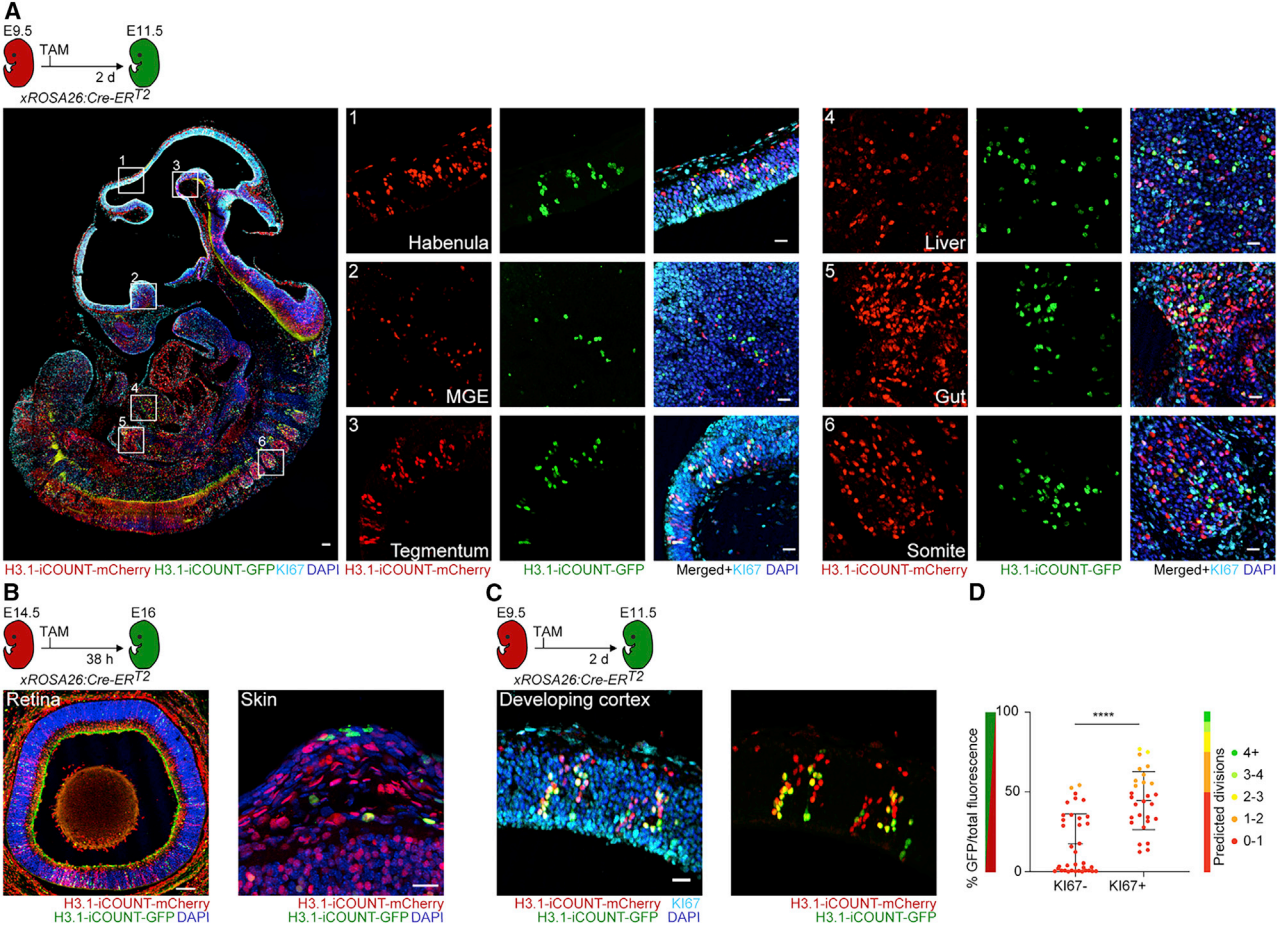
iCOUNT visualizes cell division history in embryonic tissues (A) Overview of iCOUNT mouse at E11.5 (recombination using TAM at E9.5). Right panels show mCherry (red), GFP (green), and KI67 (light blue) signals in tissues indicated. (B) Overview of the developing retina and skin in iCOUNT embryos induced with TAM at E14.5 and analyzed at E16. (C) iCOUNT-targeted cells in the developing cortex. (D) Graph shows quantification of green/total fluorescence and predicted division numbers of cells grouped by KI67 expression (mean ± SD). Nuclei were counterstained with DAPI (blue). n ≥ 28 cells derived from ≥ 3 embryos (D). ^∗∗∗∗^p < 0.0001. Images were stitched. Scale bars represent 100 μm in (A) and (B) (retina), and 20 μm in (A, right panels), (B) (skin), and (C). See also Figure S3.

To make use of the iCOUNT system for the analysis of cell division histories *in vivo*, we generated H3.1-iCOUNT knockin mice. Due to duplications of the H3.1 locus (Wang et al., 1996), mice showed mosaicism for expression of tagged histones, with approximately 71.4% of homozygous iCOUNT mice showing labeled expression of H3.1 histones (cohort of 21 simultaneously perfused, age-matched mice). First, the iCOUNT mouse was crossed to a mouse line ubiquitously expressing inducible Cre recombinase (ROSA26:Cre-ER^T2^; (Ventura et al., 2007). Mouse embryos were analyzed at embryonic day (E) 11.5 or E15 after Cre was induced using TAM injection at E9.5 or E13.5, respectively (Figures 3A and S3A). Green cells were observed in all developing organs including the developing brain, liver, gut, somites, eyes, and skin (Figures 3A and 3B). Expression of tagged H3.1 did not affect NSPC maintenance (as measured by SOX2-covered cortical area), proliferation (as measured by KI67-covered cortical area), apoptosis (as measured by cleaved Caspase-3 positive cells), or histone H3 phosphorylation (as measured by pH3-covered ventricular area; Figure S3B). Fluorescently labeled histones were symmetrically segregated during mitosis, and green/red ratios were preserved after fixation and antibody-based amplification (Figure S3C and S3D). Focusing on the developing neocortex at E11.5, columns of green cells were observed, and within those, the percentage of green fluorescence (green/total fluorescence) was increased (2.5-fold) in actively dividing cells (KI67+; Figures 3C and 3D), indicating that the iCOUNT signal reports cell division events *in vivo*.

**Figure 4:**
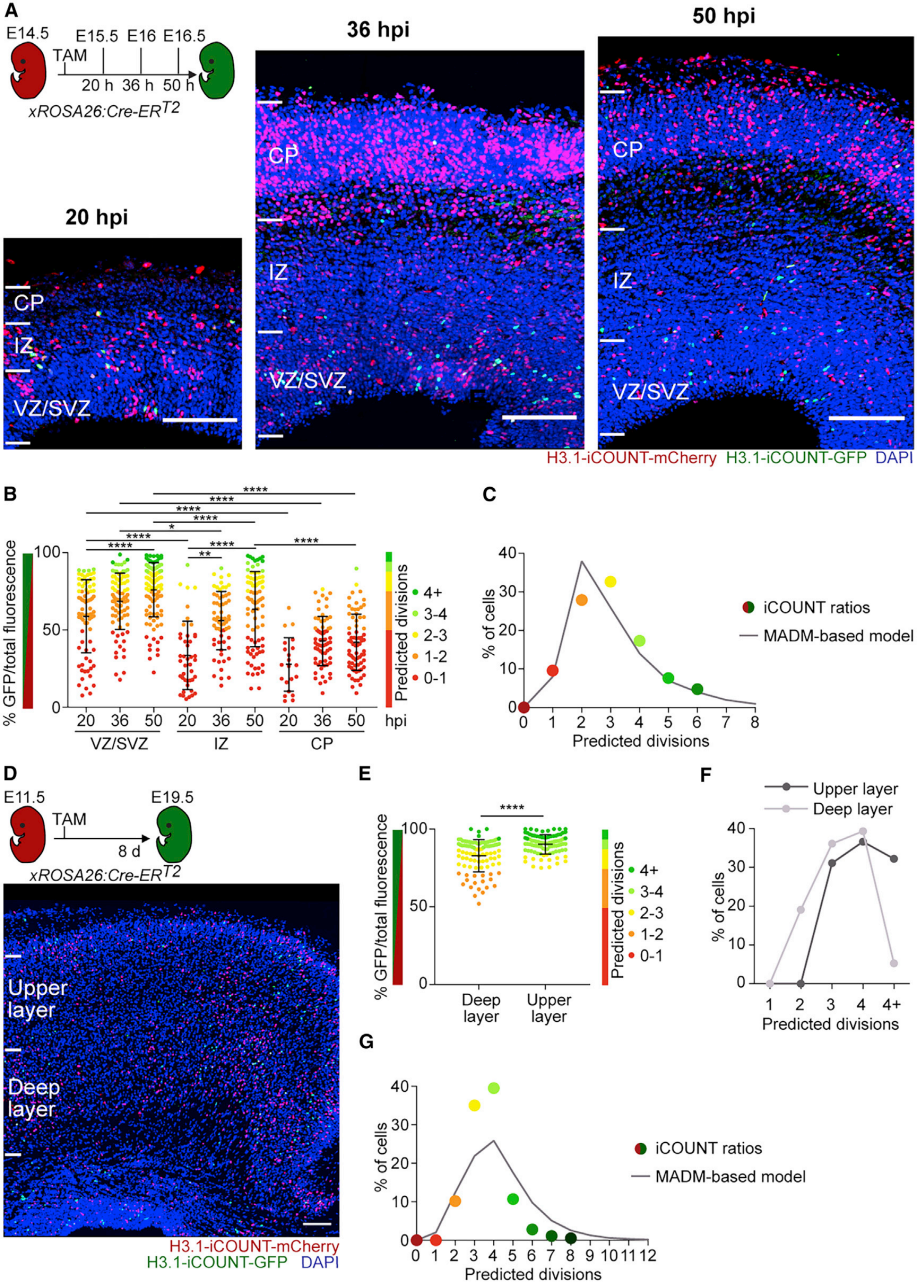
iCOUNT allows counting of previous cell divisions in the developing mouse brain (A) iCOUNT-targeted cells in the developing cortex recombined at E14.5 and analyzed at the indicated time points. (B) Quantification of green/total fluorescence and predicted division numbers of cells in the VZ/SVZ, IZ, and CP at different time points (mean ± SD). (C) Percentage of cells with iCOUNT-predicted cell divisions compared to MADM-based modeling data (Gao et al., 2014) in the VZ/SVZ 50 hours post-induction. (D) iCOUNT-targeted cells in the developing cortex recombined at E11.5 and analyzed at E19.5. (E) Quantification of green/total fluorescence and predicted division numbers of cells in the deep and upper cortical layers (mean ± SD). (F) Percentage of cells with predicted cell division numbers in upper (dark gray) and deep (light gray) cortical layers. (G) Percentage of cells with iCOUNT-predicted cell divisions compared to MADM-based modeling data (Gao et al., 2014) in cortical layers (E11.5 to E19.5 chase). Nuclei were counterstained with DAPI (blue). n ≥ 21 (B) and n ≥ 40 (E) cells derived from ≥ 3 embryos. ^∗^p < 0.05; ^∗∗^p < 0.01; ^∗∗∗∗^p < 0.0001. Images were stitched. Scale bars represent 100 μm. See also Figure S4.

Next, Cre recombinase was induced at E14.5, and the ratio of red/green fluorescence was analyzed in cells within different areas of the developing cortex and at different time points after Cre induction (E15.5, E16, and E16.5; Figure 4A). Strikingly, the percentage of green fluorescence increased with time after Cre induction, with an initial increase in the ventricular and subventricular zone (VZ/SVZ), where radial glia cells are constantly dividing, and later in the intermediate zone (IZ), and lastly in the cortical plate (CP; Figures 4B, S4A, and S4B) (Götz and Huttner, 2005). These data suggest that radial glia cells divide between zero and >4 times within 50 hours, generating neurons with gradually increasing fractions of green fluorescence that migrate into the IZ and later to the CP. These findings are consistent with previous estimates of radial glia cell divisions (Figure 4C; "Theory" in STAR Methods; Methods S1) (Gao et al., 2014). We next used the iCOUNT system to test the hypothesis that the same radial glia cells first generate deep-layer and, later, upper-layer neurons within the cortex (Beattie and Hippenmeyer, 2017). Cre was induced at the start of neurogenesis (E11.5), and cortices were analyzed after the completion of cortical neurogenesis (E19.5). iCOUNT data revealed an increase in the percentage of green fluorescence in cells within the upper layers compared to the deep layers (Figures 4D and 4E). However, we observed an overlap between the two layers (cells with 3 or 4 previous divisions contribute 36% or 39% to deep layer and 31% or 36% to upper layer; Figure 4F). iCOUNT identifies overall similar numbers (peaking at 3 to 4 cell divisions) compared to previously reported mathematical modeling data derived from Mosaic Analysis with Double Markers (MADM)-based clonal data (Figure 4G; Methods S1). However, the overlap of previous cell divisions in deep and upper layers suggests that not all radial glia cells follow a temporally fixed program of set cell divisions, thus challenging previous snapshot-based estimates of cell division biographies of radial glia cells and their progeny in the embryonic brain (Gao et al., 2014; Götz and Huttner, 2005).

**Figure 5:**
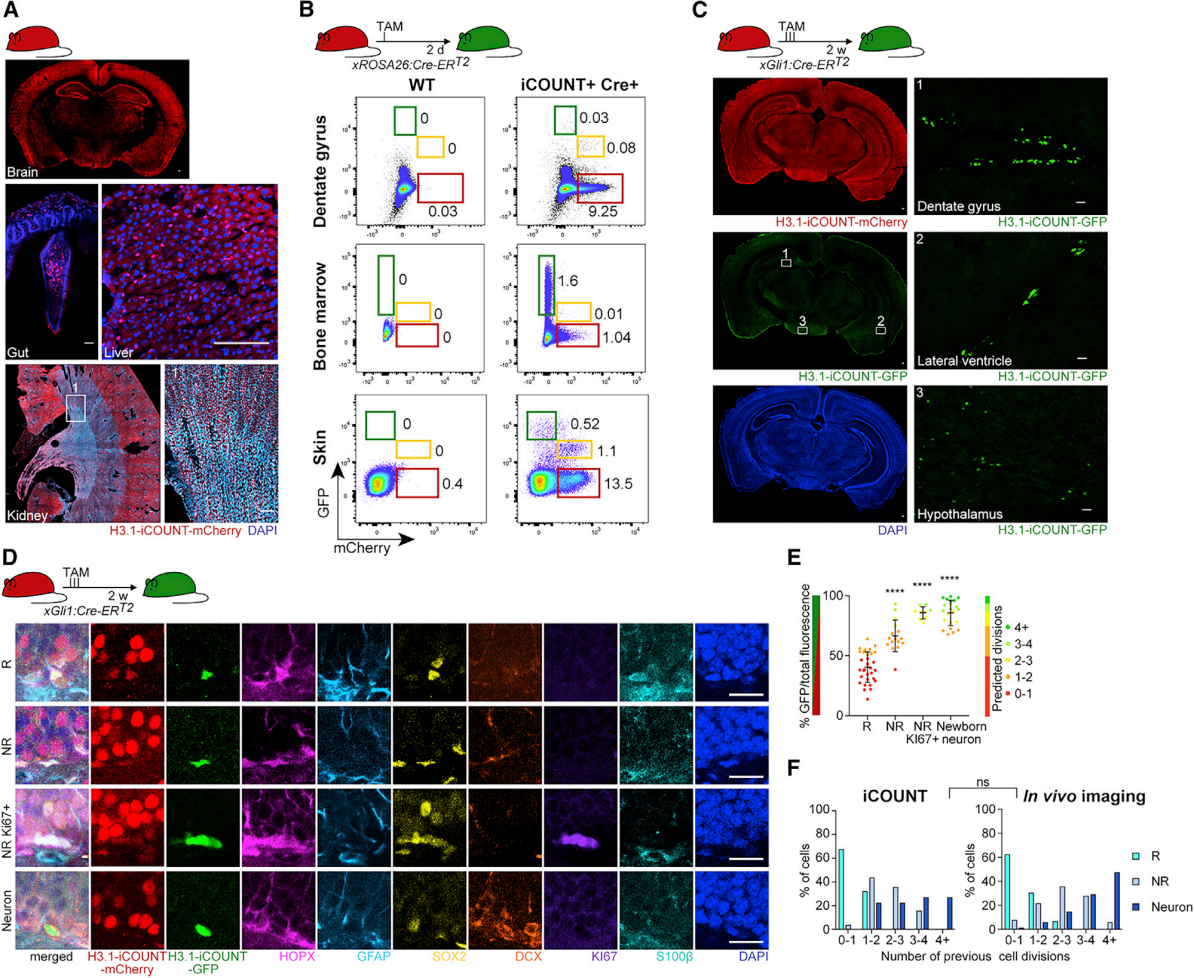
iCOUNT signal in adult mouse tissues (A) Overview of an adult iCOUNT mouse with expression of the tagged H3.1 in diverse tissues as indicated. (B) FACS analyses of cells with distinct red/green histone ratios in diverse tissues as indicated. Left panel shows controls, and right panel shows cells expressing newly synthesized (green) histones after inducible Cre-based recombination. (C) Conditional recombination in adult NSPCs identifies iCOUNT-targeted cells in the adult brain. Left panels show overview, and boxed areas are shown in higher magnification (right panels). (D) 4i-based phenotyping of Cre-targeted cells in the adult hippocampus using a panel of protein markers as indicated reveals radial glia-like NSPCs (R), non-radial glia-like NSPCs (NR), actively dividing NR (KI67+), and newborn neurons. (E) Quantification of green/total fluorescence and predicted division numbers of cells in the adult hippocampus 2 weeks after Cre-based recombination (mean ± SD). (F) Number of previous cell divisions based on iCOUNT and chronic intravital imaging. Note the comparable distribution among R, NR, and newborn neurons. Nuclei were counterstained with DAPI (blue). n ≥ 10 cells derived from ≥ 3 mice (E). ^∗∗∗∗^p < 0.0001, ns, not significant. Images were stitched. Scale bars represent 100 μm in (A) and (C) and 20 μm in (C, right panels) and (D). See also Figure S5.

To use the iCOUNT in adult mice, we examined the expression of H3.1-iCOUNT in different organs and found mCherry positive cells in all tissues analyzed (shown are brain, gut, liver, and kidney; Figure 5A). Crossing H3.1-iCOUNT mice to ROSA26:Cre-ER^T2^ mice allowed the analysis of red/green cells in different organs. Using FACS analysis 2 days after Cre induction, we observed red/green cells—for example, in the brain, bone marrow, and skin (Figure 5B)—compatible with the existence of dividing stem cells in those niches and indicating the broad usability of H3.1-iCOUNT mice. To focus on adult NSPCs, iCOUNT mice were crossed with mice expressing inducible Cre recombinase under the Gli1 promoter (Gli1:Cre-ER^T2^), leading to the expression of Cre selectively in adult NSPCs (Ahn and Joyner, 2005). Two weeks after TAM-induced Cre-mediated recombination, red/green cells were mostly observed in two regions, the dentate gyrus (DG) and the SVZ, with only few cells labeled in the hypothalamus, in agreement with previous literature reporting neurogenic zones in the adult brain (Figure 5C) (Denoth-Lippuner and Jessberger, 2021). Using iterative immunostaining technology (4i), we performed immunofluorescent labeling of different marker proteins, besides the iCOUNT colors in red and green, to phenotype recombined cells (Figures 5D, S5A, and S5B) (Gut et al., 2018). We used antibodies detecting HOPX, SOX2, GFAP, DCX, IBA1, NEUN, KI67, S100β, and OLIG2 on whole brain sections to distinguish different cell types in the DG: radial glia-like (R) cells (HOPX+, SOX2+, GFAP+, with radial process); non-radial glia-like (NR) cells (HOPX+, SOX2+, without radial process); dividing NR cells (HOPX+, SOX2+, KI67+); newborn neurons (DCX+); mature neurons (NEUN+); astrocytes (SOX2+, GFAP+, S100β+); oligodendrocytes (OLIG2+); and microglia (IBA1+) (Figures S5A and S5B) (Berg et al., 2019). As expected, most iCOUNT green cells were categorized as R, NR cells, or newborn neurons (Figure 5D). Within those, R cells showed the lowest percentage of green fluorescence that increased in NR cells and that was further elevated in actively dividing (KI67+) NR cells and newborn neurons (Figure 5E). We used iCOUNT to predict cell division histories of individual cells derived from R cells in the adult neurogenic lineage and found no statistically significant differences compared to cell division numbers obtained by intravital imaging (Figure 5F) (Bottes et al., 2021). The expression of iCOUNT did not affect the number of R cells, proliferating NR cells, newborn neurons, or apoptotic cells (as measured by cleaved Caspase-3 positive cells; Figure S5C). To compare the ability of iCOUNT to determine NSPC divisions with previously used H2B-GFP overexpressing mice, Dox-inducible H2B-GFP mice were induced for 16 days and either analyzed immediately or after 27 days of chase (Foudi et al., 2009; Furutachi et al., 2015; Morcos et al., 2020). H2B-GFP fluorescence was relatively stable after loading and following the chase within the DG, given that the DG mostly consists of postmitotic neurons (Figure S5D). However, the heterogeneity of H2B-GFP expression after loading masked a potential dilution of H2B-GFP in cells with proliferative potential (e.g., dividing R and NR cells), due to the fact that cells could have undergone up to four divisions and would have still remained within the range of variation (Figures S5D and S5E). In contrast, iCOUNT-derived numbers closely matched cell division estimates of R, NR cells, and neurons using chronic *in vivo* imaging approaches 2 weeks after Gli1-dependent reporter expression (Figure 5F), thus cross-validating adult brain intravital imaging and iCOUNT-based technologies (Bottes et al., 2021). Together, the iCOUNT data confirmed that R cells divide few times in the adult brain, whereas NR cells substantially amplify the pool of neural progenitor cells that subsequently differentiate into neurons originating from a range of mother cell divisions (Bonaguidi et al., 2011; Pilz et al., 2018).

### Using iCOUNT in human ESCs and forebrain organoids

**Figure 6:**
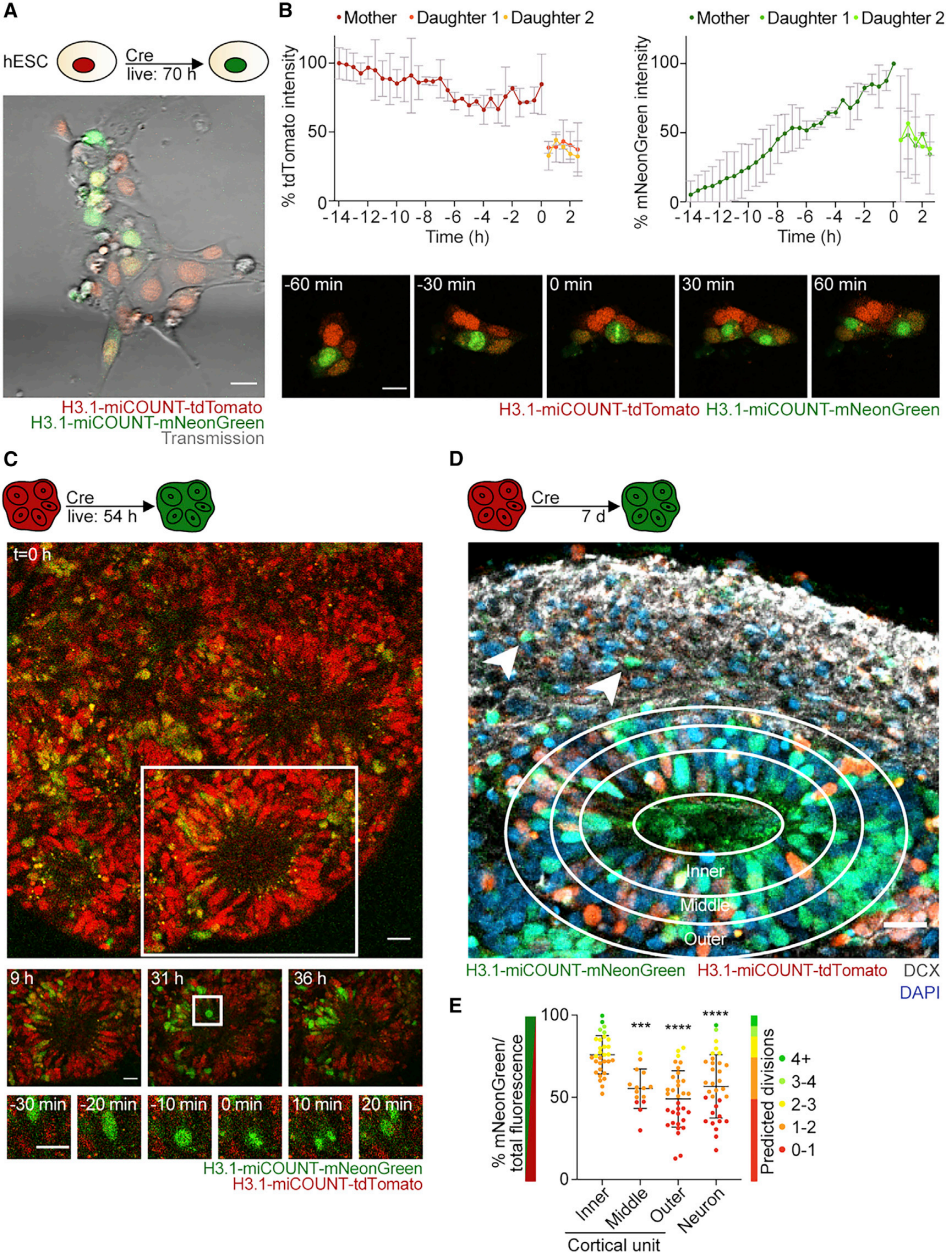
miCOUNT signal in human forebrain organoids (A) Selected time point of live imaging for 70 hours after induction with Cre, showing tdTomato (red)- and mNeonGreen (green)-tagged histones in human ESCs. (B) Graph shows measured changes in fluorescence intensities for red (tdTomato) and green (mNeonGreen) histones of single cells aligned by the time of division (mean ± SD). Lower panel shows images of a dividing human ESC with high temporal resolution. (C) Selected time points of live imaging of human miCOUNT expressing cells in a forebrain organoid for 54 hours. Middle panels show high magnifications of the cortical unit boxed in the top panel. Lower panels show high magnifications of a dividing progenitor boxed in the middle panel. (D) Micrograph of miCOUNT cells 7 days after Cre-based recombination shows increase in mNeonGreen (green) histones in the inner parts of the cortical unit (circles depict analyzed areas). Neurons are stained with DCX (white, arrow heads point toward examples of neurons). (E) Quantifications of green/total fluorescence and predicted division numbers of cells in human organoids in distinct areas of a cortical unit as indicated (mean ± SD). Nuclei were counterstained with DAPI (blue). n ≥ 3 cells (B); n ≥ 15 cells derived from ≥ 3 organoids (E). ^∗∗∗^p < 0.001; ^∗∗∗∗^p < 0.0001. Images were stitched. Scale bars represent 20 μm. See also Figure S6 and Video S3.

To study individual cell division histories in human cells, we first tagged H3.1 with miCOUNT in human embryonic stem cells (hESCs) and followed single cells using time-lapse microscopy after Cre, observing the predicted color exchange also in human cells (Figures 6A and 6B). Thus, we generated human forebrain-regionalized organoids from miCOUNT hESCs (Qian et al., 2016). Organoids (23 days in culture) were first injected with Cre-expressing plasmids, and 24 hours later, live whole organoid imaging was performed for 54 hours. Visualization of single cells within an intact organoid revealed the expected color exchange and symmetric segregation of miCOUNT tagged histones (Figures 6C, S6A, and S6B; Video S3). Next, we performed 4i on organoids and found the highest percentage of green fluorescence in the inner part of cortical units compared to the middle, outer parts, and newly generated neurons (Figures 6D, 6E, and S6C). To test for dual color switch in hESCs, ER^T2^-Cre-ER^T2^ expressing NUP155-miCOUNT hESCs were generated, treated for 2 days with TAM, and then electroporated with Flp recombinase. A color exchange from red (tdTomato) to green (GFP) and then from green to blue (tagBFP) was observed both by fluorescent imaging and FACS (Figure S6D and S6E).

Video S3. Human-forebrain-regionalized organoids derived from H3.1-miCOUNT expressing hESCs were imaged for 54 h starting 24 h post-Cre injection, related to Figure 6 A gradual change from red (tdTomato) to green (mNeonGreen) can be seen in cells that received Cre. Arrow highlights a H3.1-mNeonGreen expressing cell that undergoes interkinetic nuclear migration and divides at the ventricle. Movie is set to slow motion during division. Duration of the movie: 54 h. Tiles were stitched. Scale bar represents 20 μm.

### Molecular consequences of previous cell division events in mouse and human NSPCs

**Figure 7:**
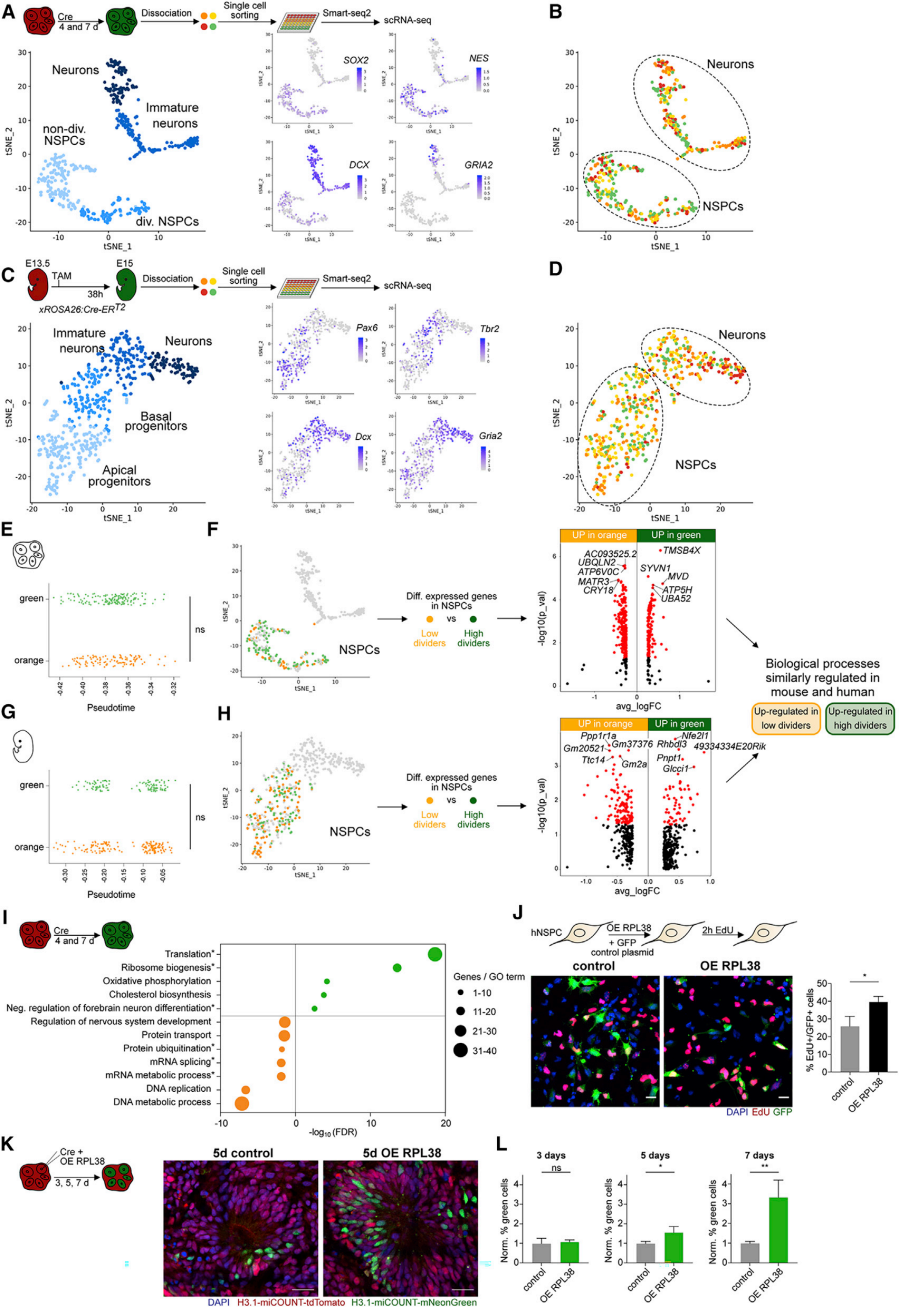
Molecular consequences of previous cell divisions in mouse and human NSPCs (A) scRNA-seq of human forebrain organoids identifies expected clusters of cells (left panel shows tSNE) with representative marker genes imposed on identified cluster (right panels). (B) Clustering of iCOUNT-targeted human cells into NSPCs and neurons. Cells with different green/total fluorescence ratios are indicated on the tSNE plot. (C) scRNA-seq of developing mouse cortex identifies expected clusters of cells (left panel shows tSNE) with representative marker genes imposed on identified cluster (right panels). (D) Clustering of iCOUNT-targeted mouse embryonic cells into NSPCs and neurons. Cells with different green/total fluorescence ratios are indicated on the tSNE plot. (E) Position of orange and green organoid-derived cells along the pseudotime axis. (F) Volcano plot showing differentially expressed genes (p < 0.05 in red) between orange and green NSPCs derived from human brain organoids. (G) Position of orange and green cortex-derived cells along the pseudotime axis. (H) Volcano plot showing differentially expressed genes (p < 0.05 in red) between orange and green NSPCs derived from the developing cortex of embryonic mice. (I) Selected “Biological Process” Gene Ontology (GO) terms enriched in cells with high (green) or low (orange) cell division history within the NSPC cluster of human organoids. Asterisks depict GO terms that were shared between human organoids and developing mouse cortex. Differently sized circles represent numbers of genes within each GO term. (J) Images of human NSPCs overexpressing RPL38 or control plasmid labeled with EdU for 2 hours. Quantifications show percentage of EdU positive cells (mean ± SD). (K) Images of a cortical unit of miCOUNT organoids 5 days post-injection of Cre-expressing plasmids in combination with RPL38 expressing or control plasmids. (L) Quantifications of the percentage of green and orange cells determined by FACS analyses normalized by the control levels (mean ± SD). Nuclei were counterstained with DAPI (blue). n ≥ 3 biological replicates (J and L). ns, not significant, ^∗^p < 0.05; ^∗∗^p < 0.01. Images were stitched. Scale bars represent 20 μm. See also Figure S7 and Table S1.

We next aimed to identify the molecular consequences of previous cell divisions on individual cells using single-cell RNA sequencing (scRNA-seq). First, we used FACS to sort single cells with different fractions of red/green fluorescence (Figure S7A) of human miCOUNT organoids 4 and 7 days after Cre-expressing plasmid injections, followed by Smart-Seq2-based scRNA-seq (Jaeger et al., 2020, Picelli et al., 2014). In line with previous organoid scRNA-seq experiments, t-distributed stochastic neighbor embedding (t-SNE) revealed four clusters, which were classified as dividing and non-dividing NSPCs, and immature and mature neurons, based on differential expression of known marker genes (Figure 7A) (Camp et al., 2015). Within each cluster, cells of different iCOUNT red/green ratios were equally distributed (Figure 7B). To compare cells of different division histories but excluding non-recombined cells, we focused on orange (low dividers) and green (high dividers) cells. They showed no difference in pseudotime progression (Figure 7E), allowing us to compare their gene expression profiles within either NSPCs or neurons (Figures 7F and S7C). In parallel, nuclei from mouse-developing cortices were collected at E15, 38 hours post-induction of Cre, and FACS sorted based on their iCOUNT ratio (Figure S7B). Again, t-SNE analysis revealed four major clusters: apical and basal progenitors, as well as immature and mature neurons, based on the expression of marker genes (Figure 7C), with cells containing different red/green ratios in all clusters (Figure 7D). Orange and green cells were equally distributed along the pseudotime axis (Figure 7G), allowing for the identification of division history-dependent, differentially expressed genes within the cluster of NSPCs or neurons (Figures 7H and S7D).

In organoids, we identified 241 genes upregulated in orange and 120 genes in green NSPCs and, in the mouse cortex, 117 and 54 genes, respectively (Figures 7F, 7H, and S7E; Table S1). Gene Ontology (GO) enrichment analysis revealed different pathways enriched in orange or green cells, of which many were also found in the GO enrichment analysis of the developing mouse cortex scRNA-seq data (asterisks in Figure 7I and filled circles in Figures S7F and S7G). Genes enriched in orange iCOUNT cells within the NSPC clusters were, for example, involved in mRNA splicing, whereas genes enriched in green cells were involved in ribosome biogenesis (Figure 7I). To test whether manipulating ribosome biogenesis affects stem cell divisions, we first overexpressed RPL38, a component of the large ribosomal subunit (Klinge et al., 2011), in human NSPCs and found that RPL38 enhances NSPC proliferation, suggesting a role for ribosomal biogenesis in stem cell activity (Figure 7J). Next, we used miCOUNT organoids that were injected with Cre-expressing plasmids together with a plasmid overexpressing RPL38 (Figure 7K). Dissociated organoids were analyzed using FACS. No difference in green to red ratio, as a measure of cell division capacity, was observed after 3 days. In contrast, we found an increased green to red ratio 5 and 7 days after overexpression of RPL38 (Figures 7L and S7H). Thus, we here discovered that cells with different individual cell division histories are distinct in their gene expression profiles and that manipulating identified pathways harbors the potential to enhance NSPC divisions.

## Discussion

Recently, substantial progress has been made to derive full lineage tree information from individual cells using barcoding techniques (Baron and van Oudenaarden, 2019; Fuentealba et al., 2015; Gao et al., 2014; Kalhor et al., 2018; Mayer et al., 2015; McKenna et al., 2016). However, potential intermediate steps are inherently hidden in these approaches, making it challenging to infer information such as which cells divided, how many times, and how many cells were born but later lost due to cell death. Several approaches were tried to overcome this problem by loading cells with CFSE or by artificially overexpressing H2B-GFP, followed by the analysis of the dilution of these labels. However, both methods have shortcomings for counting cell divisions that are remedied by the iCOUNT system in the following ways: (1) after loading cells with CFSE or H2B-GFP, the variability of starting fluorescence only allows for a dilution analysis on a population level. Moreover, leaky background expression of the repressed H2B-GFP transgene further complicates accurate data interpretation (Challen and Goodell, 2008, Morcos et al., 2020). In contrast, iCOUNT-based strategies enable the quantification of fluorescent ratios, thus eliminating variations based on differential expression, cell size, and imaging conditions, and thereby enabling a precise counting of cell divisions on a single cell level. (2) By tagging the stable proteins in non-dividing cells (H3.1 or NUP155; (Toyama et al., 2013, 2019), the iCOUNT shows higher stability in non-dividing cells compared to CFSE or overexpressed H2B-GFP. (3) Whereas CFSE is only applicable to cells *in vitro* or specific stem-cell niches (Takizawa et al., 2011; Telley et al., 2016; Vannini et al., 2016), iCOUNT is ubiquitously expressed in the whole mouse. However, H3.1 tagging may cause mosaic expression of the RITE cassette, as there are 9 different genes encoding for the histone-variants H3.1 and H3.2 within the *HIST1* gene cluster on chromosome 13 (Wang et al., 1996). Indeed, we observed mosaic expression, and approximately 28.6% of mice that had the RITE cassette correctly inserted (verified via genomic PCR) did not express the transgene. (4) H2B-GFP alters cellular properties due to artificial histone overexpression and affects nucleosome structure and function, preventing its compatibility with transcriptomics and altering behavior of these mice (Challen and Goodell, 2008; Ito et al., 2014; Meeks-Wagner and Hartwell, 1986; Ricci et al., 2015; Toyama et al., 2013, 2019; Tumbar et al., 2004). In contrast, iCOUNT is inserted into the endogenous locus and does not lead to overexpression of the gene, exemplified here by using H3.1 and NUP155. (5) In contrast to previously described H2B-GFP-based approaches where cells are ubiquitously loaded with H2B-GFP, iCOUNT can be combined with clonal lineage tracing approaches using low doses of Cre recombinase. Thus, iCOUNT allows for the precise counting of cell divisions in complex tissues and the analysis of molecular consequences of previous rounds of cell divisions in single cells.

We show the accuracy of iCOUNT using *in vitro* time-lapse imaging where the measured values significantly differ between 0, 1, 2, 3, and 4 cell divisions and are highly similar to what is expected theoretically. Measured values were slightly less “green” than predicted, most probably due to a delay between transcription of H3.1-GFP and the detection of the protein. Due to experimental limitations, only 4 consecutive cell divisions were recorded; however, a slight decrease in accuracy was observed in the 4^th^ division. Therefore, *in vivo* estimates were limited to 1 to 4 cell divisions. Comparing cell division estimates obtained from NSPCs using iCOUNT to data that were obtained by chronic intravital imaging and MADM-based modeling further supports the counting accuracy of iCOUNT (Bottes et al., 2021; Gao et al., 2014). We were able to determine the number of previous cell divisions of radial glia cells, and thereby experimentally confirmed the previous mathematical modeling-based predictions (Gao et al., 2014). We thereby demonstrate the usability of the iCOUNT system both *in vitro* and *in vivo*.

Comparing the division history of neurons in the deep and upper layer of the developing mouse cortex revealed that, indeed, radial glia cells generating deep layer neurons divided less as compared to those generating upper layer neurons, supporting the current model (Gao et al., 2014). However, we observed a substantial amount of deep- and upper-layer neurons that showed the same green/red ratio and thus arose from radial glia cells that underwent the same amount of cell divisions. These findings indicate that not all radial glia cells first produce deep and, later, upper layer neurons, but rather suggests the existence of different radial glia cells being active at different times during development. Further characterization using iCOUNT embryos at different time points will render important new insights into the division pattern of single radial glia cells during cortex development in mice.

Important insights into the division potential and distinct responsiveness of stem cells have been derived from intravital imaging (Barbosa et al., 2015; Gurevich et al., 2016; Lo Celso et al., 2009; Pilz et al., 1998; Ritsma et al., 2014; Rompolas et al., 2012; Xie et al., 2009). However, chronic imaging is not possible in a variety of stem cell niches and not easily compatible with methods to isolate cells for molecular phenotyping. In contrast, we were able to isolate hundreds of cells with individual division histories from various organs simultaneously and analyze their molecular profiles using scRNA-seq.

As proof of principle, we performed scRNA-seq of mouse developing cortex and human forebrain organoids. Orange cells were compared to green cells; as for red cells, it remains elusive whether they were non-dividing or did not recombine. We discovered networks of differentially expressed genes depending solely on the cell division history of the cells. Importantly, several of them were conserved between mouse and human cells. Among the common pathways upregulated in low-dividing NSPCs are the mRNA metabolic process, mRNA splicing, and protein ubiquitination. Future experiments will reveal whether, for example, increased protein ubiquitination is observed due to increased protein degradation or even aggregation of certain proteins (Galves et al., 2019). On the other hand, several pathways were upregulated in high-dividing NSPCs in mouse and human NSPCs: translation, ribosome biogenesis, and negative regulation of forebrain neuron differentiation. Whereas the latter is a sign for stem cell maintenance, despite a high number of divisions, ribosome biogenesis had been implicated in cell growth and cancer (Mayer and Grummt, 2006; Pelletier et al., 2018).

To test for the biological significance of the identified pathways, we artificially elevated the levels of ribosomal biogenesis by overexpressing *RPL38* in human NSPCs and organoids. Indeed, we observed increased proliferation of human NSPCs in adherent monolayer cultures and in brain organoids after experimentally increasing the levels of a single ribosomal protein, RPL38, that has been previously associated with HOX gene regulation and cell division in cancer cells (Ji and Zhang, 2020; Kondrashov et al., 2011; Xue et al., 2015). RPL38 function in organoids was analyzed using FACS of miCOUNT organoids, highlighting the potential of the miCOUNT system to screen for genes and pathways that may cause enhanced or reduced stem cell proliferation.

Furthermore, comparing the expression profiles of different iCOUNT-colored cells derived from other somatic stem cell compartments will render both stem-cell-specific and global effects of an individual cell’s division history. This will greatly broaden our understanding of behavioral and molecular consequences of previous cell divisions of different cell types in mammalian stem cell niches.

The miCOUNT approach will not only allow the exact counting of more divisions (as validated using long-term microscopy) but also allows for the visualization of stem cell division patterns during two different time points; for example, during development and in the adult organism. Choosing different time windows of induction with Cre and Flp will render important information about cell division patterns of individual stem cells at various time points. Together, determining the cell division biography of individual cells throughout their lifespan and its molecular consequences will further our understanding of stem cell behavior in health and disease.

### Limitations of the study

The molecular and cellular consequences of previous cell divisions remain to be fully characterized and functionally tested in mouse and human NSPCs and other tissues. In addition, unwanted molecular effects of tagging stable, cell-cycle-dependent proteins such as histone H3.1 and the nuclear pore component NUP155 within genetically targeted cells cannot be ruled out and may need further *in vitro* and *in vivo* analyses of iCOUNT/miCOUNT-modified tissues and future optimizations.

## STAR★Methods

### Key resources table

**Table:** 

REAGENT or RESOURCE	SOURCE	IDENTIFIER
**Antibodies**		

mCherry	Thermo Fisher Scientific	Cat No. PA5-34974
		RRID:AB_2552323
GFP	Rockland	Cat No. 600-101-215
		RRID:AB_218182
KI67	eBioscience	Cat No. 14-5698-82
		RRID:AB_10854564
HOPX	Santa Cruz	Cat No. sc-398703
		RRID:AB_2687966
GFAP	Novus	Cat No. NBP1-05198
		RRID:AB_1556315
SOX2	Invitrogen	Cat No. 14-9811-82
		RRID:AB_11219471
S100β	Abcam	Cat No. ab52642
		RRID:AB_882426
DCX	Santa Cruz	Cat No. sc8066
		RRID:AB_2088494
DCX	Millipore	Cat No. AB2253
		RRID: AB_1586992
OLIG2	Millipore	Cat No. AB15328
		RRID:AB_2299035
IBA1	Wako	Cat No. 019-19741
		RRID:AB_839504
NEUN	Millipore	Cat No. MAB377
		RRID:AB_2298772
SOX2	R and D Systems	Cat No. MAB2018
		RRID:AB_358009
KI67	BD Biosciences	Cat No. 550609
		RRID:AB_393778
TBR2	Abcam	Cat No. ab23345
		RRID:AB_778267
PAX6	Covance	Cat No. PRB-278P
		RRID:AB_291612
CTIP2	Abcam	Cat No. ab18465
		RRID:AB_2064130
TUJ1	Covance	Cat No. MMS-435P
		RRID:AB_2313773
SOX2	eBioscience	Cat No. 14-9811-82
		RRID:AB_11219471
TBR2	eBioscience	Cat No. 14-4875-82
		RRID:AB_11042577
TBR2	Abcam	Cat No. ab183991
		RRID:AB_2721040
SOX2	Santa Cruz	Cat No. sc17320
		RRID:AB_2286684
Cleaved Caspase-3 (Asp175)	BioConcept	Cat No. 9664S
		RRID: AB_2070042
Phosphorylated H2A.X (Ser139)	BioLegend	Cat No. 613401
		RRID: AB_315794
Phosphorylated H3 (Ser10)	Abcam	Cat No. 14955
		RRID: AB_443110

**Bacterial and virus strains**		

DH5-alpha High Efficiency	NEB	Cat No. C2987

**Biological samples**		

Mouse ESC line E14TG2a	Isolated from mouse strain 129P2/OlaHsd	N/A
	Hooper et al., 1987	
Mouse dentate gyrus NSCs	Isolated from mouse strain C57BL/6	N/A
	Knobloch et al., 2017	
Human ESC line H9	Wicell	Cat No. WA09-PCBC
Mouse strain C57BL/6JRj	Janvier	Cat No. C57BL/6JRj
		RRID:MGI:2670020

**Chemicals, peptides, and recombinant proteins**		

EGF	Thermo Fisher Scientific	Cat No. PHG0313
FGF-2	Peprotech	Cat No. 100-18B
Heparin	Sigma Aldrich	Cat No. H3149
DMEM/F-12 GlutaMAX	Thermo Fisher Scientific	Cat No. 31331028
N-2 Supplement	Thermo Fisher Scientific	Cat No. 17502048
B27-Supplement	Thermo Fisher Scientific	Cat No. 17504044
CHIR99021	STEMCELL Technologies	Cat No. 72052
LIF	Polygene	Cat No. PG-A1140-0050
PD0325901	STEMCELL Technologies	Cat No. 72182
Antibiotic-Antimycotic (Anti-Anti)	Thermo Fisher Scientific	Cat No. 15240062
mTeSR 1	Stem Cell Technologies	Cat No. 85850
mTeSR Plus	Stem Cell Technologies	Cat No. 85825
Matrigel	Corning	Cat No. 354277
Phenol Red-free growth factor reduced Matrigel	Corning	Cat No. 356238
Phenol Red-free DMEM:F12	Thermo Fisher Scientific	Cat No. 21041025
ReLeSR	Stem Cell Technologies	Cat No. 05872
ROCK Inhibitor Y-27632	Stem Cell Technologies	Cat No. 72302
Accutase	Sigma Aldrich	Cat No. A6964
CryoStor cell cryopreservation media	Sigma Aldrich	Cat No. C2874
Anti-Adherence Rinsing Solution	Stem Cell Technologies	Cat No. 07010
mTeSR-E5	Stem Cell Technologies	Cat No. 05916
Dorsomorphin	Sigma Aldrich	Cat No. P5499
A83-01	Tocris	Cat No. 293
poly-L-ornithine	Sigma Aldrich	Cat No. P3655
Laminin	Sigma Aldrich	Cat No. L2020
poly-D-lysine	Sigma Aldrich	Cat No. P6407
BMP4	R&D	Cat No. 5020-BP
G418 sulfate (Geneticin Selective Antibiotic)	GIBCO	Cat No. 10131035
Puromycin Dihydrochloride	GIBCO	Cat No. A1113802
Tamoxifen	Sigma Aldrich	Cat No. T5648
4-Hydroxytamoxifen	Sigma Aldrich	Cat No. T176
Doxycycline hyclate	Sigma Aldrich	Cat No. 44577
Doxycycline chow	Ssniff Spezialitäten	Cat No. A112D72003
DNase I	Sigma Aldrich	Cat No. 10104159001
Gelatin	Sigma Aldrich	Cat No. G1890
TrypLE	Thermo Fisher Scientific	Cat No. 12604013
Gibson Assembly Master Mix	NEB	Cat No. E2611
Zombie Violet	Biolegend	Cat No. 423113
Zombie NIR	Biolegend	Cat No. 423105
5-ethynyl-2′deoxyuridine (EdU)	Thermo Fisher Scientific	Cat No. E10187
Corning cell strainer (70 μm)	Sigma Aldrich	Cat No. CLS431751
Corning cell strainer (40 μm)	Sigma Aldrich	Cat No. CLS431750
Fetal Bovine Serum (FBS)	Sigma Aldrich	Cat No. F7524
Phusion Polymerase	NEB	Cat No. M0530L
HindIII	NEB	Cat No. R0104
SacII	NEB	Cat No. R0157
Lipofectamine 2000 Transfection Reagent	Thermo Fisher Scientific	Cat No. 11668027
Para-formaldehyde (PFA)	Sigma Aldrich	Cat No. 441244
4’,6-diamidino-2-phenylindole (DAPI)	Sigma Aldrich	Cat No. D9542
Hoechst 33342	Thermo Fisher Scientific	Cat No. H3570
NaH_2_PO_4_	Sigma Aldrich	Cat No. S3139
Na_2_HPO_4_	Sigma Aldrich	Cat No. 255793
N-Acetyl-Cysteine	Sigma Aldrich	Cat No. A9165
Glycine	Sigma Aldrich	Cat No. 50046
Urea	Sigma Aldrich	Cat No. U1250
Guanidine Hydrochloride	Sigma Aldrich	Cat No. G4505
TCEP-HCL	Sigma Aldrich	Cat No. C4706
KCL	Sigma Aldrich	Cat No. P9333
MgCl2	Sigma Aldrich	Cat No. M8266
TrisHCl	Sigma Aldrich	Cat No. T3253
Dithiothreitol	Sigma Aldrich	Cat No. D0632
Triton	Sigma Aldrich	Cat No. 93443
Protease inhibitors (cOmplete, EDTA-free)	Roche	Cat No. 4693159001
RNase inhibitors (RNasin)	Promega	Cat No. N2111
RBC Lysis Buffer	BioLegend	Cat No. 420301
SuperScript II Reverse Transcriptase	Thermo Fisher Scientific	Cat No. 18064022
KAPA HiFi HotStart ReadyMix	Kapa	Cat No. KK2600
Agencourt AMPure XP beads	Beckman Coulter	Cat No. A63882
Nextera XT DNA Library Preparation Kit	Illumina	Cat No. FC-131
EDTA	Sigma Aldrich	Cat No. EDS
DPBS	Thermo Fisher Scientific	Cat No. 14190-094
PBS	Thermo Fisher Scientific	Cat No. 10010-015
DPBS, no calcium, no magnesium	Thermo Fisher Scientific	Cat No. 14190144
Sucrose	Sigma Aldrich	Cat No. 84100
OCT compound	Sakura	Cat No. 25608-930
Donkey Serum	Millipore	Cat No. S30
ImmuMount	Thermo Fisher Scientific	Cat No. 9990402
Pentobarbital (Esconarkon®)	Streuli	Cat No. 1370110A
Hydroxyurea	Sigma Aldrich	Cat No. H8627-5G

**Critical commercial assays**		

Mouse Neural Stem Cell Nucleofector Kit	Lonza	Cat No. VPG-1004
CellTrace CFSE Cell Proliferation Kit, for flow cytometry	Thermo Fisher Scientific	Cat No. C34570
Click-iT EdU AF647 Flow Cytometry Assay Kit	Thermo Fisher Scientific	Cat No. C10419
Click-iT EdU Cell Proliferation Kit for Imaging, AF647	Thermo Fisher Scientific	Cat No. C10340
TruSeq RNA Library Prep Kit	Illumina	Cat No. RS-122-2001
TruSeq Stranded mRNA	Illumina	Cat No. 20020594

**Deposited Data**		

scRNA-seq data from iCOUNT mouse developing cortex and miCOUNT human forebrain organoids; bulk RNA-seq from wt and iCOUNT mouse ESCs	GEO: GSE167375	https://www.ncbi.nlm.nih.gov/geo/query/acc.cgi?acc=GSE167375

**Experimental models: Cell lines**		

Mouse ESCs H3.1-iCOUNT	*This manuscript*	N/A
Mouse ESCs NUP155-iCOUNT	*This manuscript*	N/A
Mouse ESCs H3.1-miCOUNT	*This manuscript*	N/A
Mouse NSCs H3.1-iCOUNT	*This manuscript*	N/A
Mouse NSCs Nup155-iCOUNT	*This manuscript*	N/A
Human ESCs H3.1-miCOUNT	*This manuscript*	N/A
Human ESCs NUP155-miCOUNT	*This manuscript*	N/A
Human ESCs ER^T2^-Cre-ER^T2^	*This manuscript*	N/A

**Experimental models: Organisms/strains**		

Mouse strain “H3iCOUNT” C57BL/6-H3c6 < tm1(mCherry,GFP)Sjes >	*This manuscript*	N/A
Mouse strain “Gli1:Cre-ER^T2^” STOCK Gli < tm3(Cre/ERT2)Alj > /J	Jackson Laboratory	Cat No. 007913
Mouse strain “ROSA26:Cre-ER^T2^” B6.129-Gt(ROSA)26Sor < tm1(Cre/ERT2)Tyj > /J	Jackson Laboratory	Cat No. 008463
Mouse strain “R26^rtTA^/Col1A1^H2B-GFP^” B6;129S4-Gt(ROSA)26Sor < tm1(rtTA^∗^M2)Jae > Col1a1 < tm7(tetO-HIST1H2BJ/GFP)Jae > /J	Jackson Laboratory	Cat No. 016836

**Oligonucleotides**		

See Table S2	*This manuscript*	N/A

**Recombinant DNA**		

pSpCas9(BB)-2A-Puro vector	Addgene	Cat No. 48139
pFA6-hphNT1	Janke et al., 2004	Euroscarf Cat No. P30347
pAAVS1-DRGFP	Addgene	Cat No. 113193
pCAG-ER^T2^CreER^T2^	Addgene	Cat No. 13777
pCAG-Cre	Addgene	Cat No. 13775
pCAG-FlpeER^T2^	Addgene	Cat No. 14756
pCAG-Flpe	Addgene	Cat No. 13787
pAAVS1-Neo-CAG-M2rtTA-H2BGFP	Addgene	Cat No. 85798
H3.1-iCOUNT homology plasmid (mouse)	*This manuscript*	N/A
NUP155-iCOUNT homology plasmid (mouse)	*This manuscript*	N/A
H3.1-miCOUNT homology plasmid (mouse)	*This manuscript*	N/A
H3.1-miCOUNT homology plasmid (human)	*This manuscript*	N/A
NUP155-miCOUNT homology plasmid (human)	*This manuscript*	N/A
ER^T2^CreER^T2^ homology plasmid (human)	*This manuscript*	N/A

**Software and algorithms**		

ApE plasmid editor	M. Wayne Davis	N/A
Fiji/ImageJ	Fiji	http://fiji.sc/
		RRID: SCR_002285
FlowJo	Tree Star	https://www.flowjo.com/solutions/flowjo
		RRID:SCR_008520
FCS Express	De Novo Software	https://denovosoftware.com/
		RRID:SCR_016431
Graphpad Prism 8	Graphpad	http://www.graphpad.com:443/
		RRID:SCR_002798
ZEN 2 Blue	Carl Zeiss	https://www.zeiss.com/microscopy/en_us/products/microscope-software/zen.html#introduction
		RRID: SCR_013672
Cutadapt v1.16	Cutadapt	https://cutadapt.readthedocs.io/
		RRID:SCR_011841
Velocyto v0.17.17	Anaconda	https://anaconda.org/bioconda/velocyto.py
		RRID:SCR_018167
Scater v1.12.2	Bioconductor	https://bioconductor.org/packages/release/bioc/html/scater.html
		RRID:SCR_015954
STAR v2.6.0c	Github	https://github.com/alexdobin/STAR
Seurat v3.1.1	Satija	https://satijalab.org/seurat/
		RRID:SCR_007322
R v.4.0.1	R Project for statistical computing	http://www.r-project.org/
		RRID:SCR_001905
Cytoscape v3.8.2	Cytoscape	https://cytoscape.org
		RRID:SCR_003032
FastICA 1.2.2	CRAN R package	https://cran.r-project.org/web/packages/fastICA/index.html
		RRID:SCR_013110

**Other**		

AggreWell 800	Stem Cell Technologies	Cat No. 34821
Ultra-Low Attachment Plate	Sigma Aldrich	Cat No. 3471
Lab Tek II chambered coverglasses	Thermo Fisher Scientific	Cat No. 155382PK
AMAXA Electroporation System	Lonza	N/A
FemtoJet 4i microinjector	Eppendorf	N/A
LSM 800 confocal microscope	Carl Zeiss	N/A
Cryostat	Microm	N/A
Cryotom	Leica	SM2010R
LSR II Fortessa	BD Biosciences	N/A
FACSAria III sorter	BD Biosciences	N/A
Mosquito robot HV genomics	TTP Labtech Ltd	N/A
Bioanalyzer	Agilent Technologies	N/A
D1000 Screen Tabe Assay	Aglient Technologies	Cat No. G2991AA
Illumina HiSeq 2500 sequencer	Illumina	N/A
Illumina HiSeq 4000 sequencer	Illumina	N/A
NanoDrop Lite Spectrophotometer	Thermo Fisher Scientific	ND-LITE

### Resource availability

#### Lead contact

Further information and requests for resources and reagents should be directed to and will be fulfilled by the Lead Contact, Sebastian Jessberger (jessberger@hifo.uzh.ch).

#### Materials availability

iCOUNT/miCOUNT mouse and human ESCs and iCOUNT-mice will be provided directly. Materials will be provided upon execution of a suitable Materials Transfer Agreement.

### Method details

#### Stem cell cultures

Mouse embryonic stem cells (mESCs; E14TG2a; (Hooper et al., 1987) were cultured in DMEM/F12 GlutaMAX medium (Thermo Fisher Scientific) supplemented with N2 and B27 (Thermo Fisher Scientific), antibiotics (Anti-Anti, Thermo Fisher Scientific), LIF (1000U/ml, Polygene), and 2i (Silva et al., 2008), PD0325901 (1 μM, STEMCELL Technologies) and CHIR99021 (3 μM, STEMCELL Technologies). mESCs were cultured in flasks coated with 0.2% gelatine (Sigma-Aldrich). Cells were regularly splitted using TrypLE (Thermo Fisher Scientific). Plasmids were introduced into cells using lipofection (Lipofectamine 2000, Thermo Fisher Scientific).

NSPCs derived from the hippocampus of adult C57BL/6 animals were cultured in DMEM/F12 GlutaMAX medium supplemented with N2, B27, antibiotics, EGF (20ng/ml, Thermo Fisher Scientific), FGF-2 (20ng/ml, PeproTech) and Heparin (5mg/ml, Sigma-Aldrich). Plasmids were introduced using the AMAXA electroporation system (Lonza) as described in (Wegleiter et al., 2019). NSPCs were differentiated into neurons by growth factor withdrawal (no addition of EGF and FGF-2) and quiescence was induced by exchanging EGF with BMP4 (Knobloch et al., 2017; Mira et al., 2010) (50ng/ml, R&D Systems).

Human embryonic stem cells (hESCs; H9 (Thomson et al., 1998), Wicell) were grown in feeder-free conditions in the absence of antibiotics. H9 hESCs were maintained in mTeSR 1 or mTeSR Plus (Stem Cell Technologies) on hESC qualified Matrigel (Corning) coated plates. Routine passaging was performed with ReLeSR (Stem Cell Technologies) to promote stemness; post-passaging, cells were maintained for 24h in media containing Y-27632 (10μM, Stem Cell Technologies). To produce single cell suspensions, hESCs were passaged with Accutase (Sigma-Aldrich). For long-term storage, hESCs were kept below −170°C in CyroStore CS10 (Sigma-Aldrich). To introduce plasmid DNA, hESCs were pretreated with mTeSR 1 or Plus containing Y-27632 for at least 2h to improve cell survival. Two million cells were resuspended in Nucleofector V (Lonza) and electroporated with an AMAXA electroporation system using program A-23 according to the manufacturer’s guidelines. All cells were kept at 37°C and 5% CO_2_. All experiments using hESCs were approved by the Kantonale Ethik-Kommission (KEK) of the Canton of Zurich, Switzerland.

#### Organoid culturing

Forebrain specific cerebral organoids were produced using the previously published protocol (Qian et al., 2016) with some alterations. At day 0, hESCs were passaged with Accutase (Sigma-Aldrich) and cells were resuspended in mTeSR 1 or Plus containing Y-27632. A cytometer was used to estimate the number of cells in the single cell suspension. The single cell suspension was added to pretreated wells AggreWell 800 (Stem Cell Technologies) that would result in 5000 cells per microwell. Pretreatment was performed with Anti-Adherence Rinsing Solution (Stem Cell Technologies) and handling of the AggreWell 800 plates was carried out following the manufacturer’s recommendations. At day 1, embryoid bodies were harvested from the AggreWells per the manufacturer’s protocol and transferred to a well of a 6 well Ultra-Low Attachment Plate (Sigma-Aldrich) containing mTeSR-E5 (Stem Cell Technologies) with Dorsomorphin (2μM, Sigma-Aldrich) and A83-01 (2μM, Tocris). Embryoid bodies were maintained in this medium until day 4, after which the protocol described (Qian et al., 2016) was followed. Organoids were either dissociated using Accutase (Sigma-Aldrich) at 37°C for 30min for FACS analyses or fixed for 30min in Paraformaldehyde (PFA, 4%, Sigma-Aldrich) and were stored in sucrose (30%, Sigma-Aldrich) until further use.

#### Gene tagging

Gene tagging using CRISPR/Cas9 was essentially performed as described (Ran et al., 2013). gRNAs were designed using https://zlab.bio/guide-design-resources and were cloned into pSpCas9(BB)-2A-Puro (Addgene No 48139) according to the protocol (Ran et al., 2013). All gRNAs used are listed in Table S2.

The H3.1-iCOUNT mouse homology plasmid was generated fusing: 405nt upstream of STOP codon of H3c6, LoxP, mCherry (without STOP codon; derived from pmCherry-N1), T2A, Neo/Kan antibiotic resistance (with STOP codon and 3′UTR, derived from pmCherry-N1), loxP, eGFP (with STOP codon and 3′UTR, derived from pEGFP-N1) and 1kb downstream and integrating it into the pFA6 (pFA6-hphNT1, Euroscarf) backbone cut with HindIII and SacII (NEB) using Gibson Assembly (NEB). The NUP155-iCOUNT homology plasmid was generated by exchanging the homology arms of H3.1-iCOUNT with 1kb upstream of STOP codon and 1kb downstream including STOP codon. The miCOUNT cassette was generated by first fusing: FRT, LoxP, tdTomato (without STOP codon), T2A, Neo/Kan antibiotic resistance (with STOP codon and 3′UTR), LoxP, mNeonGreen (with STOP codon and 3′UTR; derived from pNCS-mNeonGreen, Allele Biotech), FRT, Cerulean (with STOP codon and 3′UTR; derived from Cerulean-N1), then adding 405nt upstream of STOP codon and 1kb downstream including STOP codon, all in the pFA6 backbone as described above. The human H3.1-miCOUNT was generated by exchanging the homology arms of the mouse H3.1-miCOUNT with the human homology sequences. Human NUP155-miCOUNT was generated using a synthesized peptide containing: a flexible linker, FRT, LoxP, tdTomato (without STOP codon), 3 Flag tags, T2A, Neo/Kan antibiotic resistance (including STOP codon and 3′UTR), LoxP, eGFP (including STOP codon and 3′UTR), FRT, tagBFP and a V5 tag (including STOP codon and 3′UTR). 1kb upstream and downstream of STOP codon of NUP155 was added and assembled in pFA6 using Gibson Assembly. The PAM sites were mutated in all homology constructs to prevent cutting. To generate the ER^T2^-Cre-ER^T2^ expressing hESC line, DR-GFP from pAAVS1-DRGFP (Addgene No 113193) was replaced by ER^T2^-Cre-ER^T2^ (derived from Addgene No 13777).

To integrate the cassettes, mESCs were co-transfected and mNSPCs/hESCs were co-electroporated with the gRNA plasmid (simultaneously expressing Cas9) and the corresponding homology plasmid. ESCs were selected for correct integration of the iCOUNT cassette using G418 sulfate (300μg/ml for mESCs and 100μg/ml for mNSPCs and hESCs; GIBCO) or Puromycin Dihydrochloride (1μg/ml; GIBCO) for integration of ER^T2^-Cre-ER^T2^ respectively, until resistant colonies were visible and all cells were dead on a control plate. Correct integration was verified using PCR and fluorescence imaging. PCRs were used to check correct integration and to distinguish between *wild-type* (WT), heterozygous and homozygous clones (or animals) using primers listed in Table S2.

#### Other plasmids and constructs

For generation of a *RPL38* overexpression construct, the cDNA sequence for human *RPL38* was extracted from Ensembl genome browser (ensemble.org). cDNA was extracted from hESCs as described (Wegleiter et al., 2019). *RPL38* cDNA was amplified by PCR (primer sequences see Table S2).

Gibson assembly was used following the manufacturer’s protocol to assemble a *CAG*-*RPL38-(GGGG3)*^*3*^*-3xFLAG* overexpression plasmid using the *CAG-GFP* retroviral vector (Addgene 16664) as backbone. A control overexpression plasmid was generated by substituting *RPL38* by a start codon, such that a (GGGGS)^3^-3xFLAG fusion protein is expressed under the CAG promoter. Bacterial amplification and purification of recombinant DNA was performed as described (Wegleiter et al., 2019).

#### Generation of H3.1-iCOUNT mice

C57BL/6 mice expressing H3.1-iCOUNT were generated by PolyGene (Switzerland). C57BL/6-derived ESCs were transfected with the iCOUNT cassette targeting H3.1 as described above and selected using G418. Single clones were picked, expanded and correct insertion was verified by PCR. Positive cells were injected into blastocysts and 6 chimeras were born. Crossing three chimeras to gray C57BL/6 females yielded heterozygous H3.1-iCOUNT positive animals. To generate homozygous H3.1-iCOUNT mice, H3.1-iCOUNT ± mice were crossed with each other. Alternatively, H3.1-iCOUNT animals were crossed to Gli1:Cre-ER^T2^ mice (Jackson Lab No 007913: (Ahn and Joyner, 2004) and ROSA26:Cre-ER^T2^ mice (Jackson Lab No 008463; (Ventura et al., 2007). Animals were always genotyped to distinguish WT, heterozygous and homozygous H3.1-iCOUNT animals (see PCR above). H3.1 tagging caused mosaic expression of the RITE cassette, as there 9 different genes encoding for the histone-variants H3.1 and H3.2. within the *HIST1* gene cluster on chromosome 13 (Wang et al., 1996). Indeed, we observed mosaic expression and mice that had the RITE cassette correctly inserted (verified via genomic PCR) but did not express the transgene.

#### Animal handling

All animal experiments were performed according to Swiss regulatory standards and approved by the Veterinary office of the Canton of Zurich. iCOUNT-derived data was compared to published intravital imaging data using Gli1:CreER^T2^/tdTom mice (Bottes et al., 2021). For comparison to H2B-GFP overexpressing animals, R26^rtTA^/Col1A1^H2B-GFP^ mice (Jackson laboratory No. 016836) were fed with Doxycycline containing chow (2000 mg/kg; Ssniff Spezialitäten) for 16 days. Animals were either euthanized immediately (no chase) or after 27 days of normal diet (chase). Brains were collected immediately afterward and fixed in 4% PFA for 48h, dehydrated in 30% sucrose for 48h and stored in PBS at 4°C thereafter. All mice were kept in individually ventilated cages together with littermates under a 12h light/dark cycle and were provided with water and food *ad libitum*.

#### Fluorescence live cell imaging

All images were acquired using a confocal microscope (LSM 800, Zeiss) equipped with an incubator box to maintain 37°C and 5% CO_2_. Prior to imaging, mESCs were transfected with Cre expressing plasmids (pCAG-Cre, Addgene No 13775) and were plated 6h later on Lab Tek II chambered coverglasses (Thermo Fisher Scientific) coated with Poly-L-ornithine (10 μg/ml, Sigma-Aldrich) and Laminin (5 μg/ml, Sigma-Aldrich). mESCs were imaged every 20min for 64h (H3.1-iCOUNT) or for 38h (NUP155-iCOUNT). mESCs expressing H3.1-miCOUNT were transfected with Cre and Flp-ER^T2^ (pCAG-FlpeERT2, Addgene No 14756) simultaneously, plated after 6h, imaged every 40min for 24h before media was exchanged to TAM (0.5 μM 4-hydroxytamoxifen, Sigma-Aldrich) containing media and imaging continued for 44h. hESCs were plated on Matrigel coated chambered coverglasses immediately after electroporation with Cre and 6h later were imaged in standard media every 30min for 70h. Media was exchanged every 24h.

To image quiescent NSPCs, iCOUNT expressing NSPCs were electroporated with Cre (1 μg/μl) or WT NSPCs were electroporated with pAAVS1-Neo-CAG-M2rtTA-H2BGFP (Addgene No. 85798) and plated on laminin coated Lab Tek II chambered coverglasses. Quiescence was induced exchanging EGF with BMP4 and doxycycline (Dox; 1 μg/ml) was added to the WT NSPCs containing pAAVS1-Neo-CAG-M2rtTA-H2BGFP to load H2B-GFP. 3 days after induction of quiescence cells were imaged for 5 days taking images every 5h. Media was changed every 24h to fresh BMP4 containing media without Dox.

Organoids (23 days in culture) were injected with Cre expressing plasmids (1 μg/μl) using a FemtoJet 4i microinjector (Eppendorf) and electroporated using the AMAXA Nucleofector device. Organoids were embedded into Phenol Red-free, growth factor reduced Matrigel (Corning) on chambered coverglasses. After polymerization of the Matrigel at 37°C for 30min, imaging media was added that comprised of “Forebrain third medium” (Qian et al., 2016) with DMEM:F12 being exchanged out for Phenol Red-free DMEM:F12 (Thermo Fisher Scientific). Images were taken every 10min for 56h and media was exchanged every 24h.

#### Live cell imaging analysis

Movies were analyzed using Fiji. Lineages of four starting cells were tracked throughout the movie. Whole nuclear fluorescence (integrated density) was measured and background was subtracted for every time point. Raw values were aligned by the first time point after cell division and by appearance of green fluorescence. Next, red and green fluorescence were normalized to the highest value measured throughout the experiment within one cell lineage and mean values (±standard deviation, n ≥ 4 cells) were calculated from all time points within one cell division. Theoretical values were plotted using the cell cycle length from the experimental data. The theoretical values for red fluorescence were set to 100 prior to the first division and dropped by half with every subsequent division (100, 50, 25 etc.) whereas the green fluorescence first increased to complete the pool of histones (from 0 to 50), dropped by half after cell division (25), increased again (to 75), dropped by half (37.5) and increased again (87.5) etc. Mean values were calculated accordingly (e.g., 0 to 50 results in a mean value of 25). GFP over total fluorescence was calculated for every time point and plotted against time starting at 0h after every cell division. Cells were grouped by the appearance of green signal leading to groups of 0-1 division, 1-2 divisions etc. The GFP over total fluorescence was then grouped into 0 to 50% (0-1 division), 50% to 75% (1-2 divisions), 75% to 87.5% (2-3 divisions) 87.5% to 93.75% (3-4 divisions) and more than 93.75 (4+ divisions). The iCOUNT-based calculated division numbers were then compared to the actual division numbers.

For the miCOUNT live imaging data, fluorescence of tdTomato, mNeonGreen and Cerulean was measured at every time point and data points of three independent lineage trees were normalized by the max of each fluorescence observed. Data points were then pooled by the first cell division mNeonGreen or Cerulean was observed, respectively, and averages (±standard deviation, n ≥ 3 cells) were plotted.

hESCs were tracked as long as possible, red and green fluorescence was measured and aligned by the time of division, normalized by the max fluorescence intensity observed and plotted over time (±standard deviation, n ≥ 3 cells).

Fluorescence intensity of quiescent NSPCs was measured in cells that were not dividing within the time course of 5 days and mean fluorescence (H2B-GFP) or mean of green/total fluorescence (iCOUNT) was plotted for every time point (±standard deviation, n = 10 cells each).

Cells depicting green fluorescence were tracked within the organoid as long as possible. Green and red fluorescence of 11 cells was measured for every time point, normalized by the max fluorescence and then plotted over time (±standard deviation, n = 11 cells). Measurements were aligned by the time of cell division. To assess the symmetric distribution of red and green fluorescence between daughter cells, the ratio of green over red fluorescence was measured in daughter cells right after division and values were pairwise plotted (n ≥ 9 cells).

Imaging of large fields of views were performed using the tile imaging function and pictures were stitched together after imaging using the plug in provided by the ZEN 2 blue software (Carl Zeiss). Stitched images were indicated in the figure legends.

#### Other cell imaging

To test the effect of increased RPL38 levels on NSPC proliferation, human NSPCs (Roche) were electroporated using 1.38 pmol per 2x10^6^ cells of control or RPL38 expression plasmid together with pmaxGFP as electroporation control in triplicates, and plated onto poly-L-ornithine (50 μg/ml) and laminin (5 μg/ml) coated coverslips. 48h after electroporation, cells were labeled for 2h with EdU (10 μM, Thermo Fisher Scientific), followed by fixation using PFA (4%) for 10min. Click-reaction was then performed using the Click-iT EdU AF647 Proliferation Kit (Thermo Fisher Scientific) and following the manufacturer’s protocol, and nuclei were counterstained with DAPI (1:1000) prior to imaging. GFP-positive and GFP/EdU double-positive cells were counted in three images per sample, mean values (±standard deviation, n ≥ 3 independent electroporations) were plotted.

miCOUNT organoids were injected with Cre expressing plasmids (0.5 μg/μl) together with control or RPL38 expression plasmid (0.5 μg/μl) and electroporated using the AMAXA nucleofector device. At 35 days in culture, 3, 5, or 7 days post injection, organoids were either fixed using 4% PFA for 30min. After fixation, organoids were transferred to 30% sucrose solution over night (ON), embedded in OCT compound and frozen using liquid nitrogen. Organoids were sectioned into 40 μm slices using a cryostat and nuclei were counterstained with DAPI (1:1000) prior to imaging.

To assess the recombination efficiency, mESCs were co-transfected with Cre (1 μg/μl) and a Cre-reporter plasmid (lox-STOP-lox mKO2, 1 μg/μl, a gift from Fumio Matsuzaki, (Shitamukai et al., 2011). 24h after transfection, mESCs were imaged and the percentage of iCOUNT-GFP positive cells was determined among the mKO2 positive cells using manual counting. Mean values (±standard deviation, n = 3 independent transfections) were plotted.

#### Immunostaining

For the embryonic cell division analyses, time-mated H3.1-iCOUNTxROSA26:CreER^T2^ mice received a single intraperitoneal injection of tamoxifen (180mg/kg, Sigma-Aldrich, dissolved in corn oil, Sigma-Aldrich) at different embryonic time points as indicated in figures. Whole embryos (at E11.5 and E15) and embryonic brains (at E15.5, E16, E16.5 and E19) were dissected and fixed overnight in 4% PFA at 4°C. They were first cryoprotected in 15% sucrose at 4°C (overnight) and then in 30% sucrose at 4°C (overnight). Tissues were frozen in OCT compound (Tissue-Tek, Sakura) and stored at −80°C until they were serially sectioned at 20μm using a cryostat (Microm). The slides with frozen sections were stained using methods as described previously (Wegleiter et al., 2019). Tissues were stained against mCherry (1:250, rabbit, Thermo Fisher Scientific), GFP (1:250, goat, Rockland), KI67 (1:250, rat, eBioscience), SOX2 (1:200, rat, eBioscience), SOX2 (1:200, goat, Santa Cruz), TBR2 (1:200, rat, eBioscience), TBR2 (1:200, rabbit, Abcam), CTIP2 (1:200, rat, Abcam) and nuclei were counterstained using 4’,6-Diamidine-2′-phenylindole dihydrochloride (DAPI, 1:5000; Sigma-Aldrich). To assess the effects of staining, mESCs were transfected with Cre, cells were fixed 48h post transfection using 4% PFA for 5min and were stained using the same antibodies against mCherry and GFP. To investigate adult tissues, animals received a lethal dose of Esconarkon (Streuli) followed by trans-cardial perfusion using first 0.9% saline and then 4% PFA in phosphate buffer (0.1 M phosphate, Sigma-Aldrich). Tissues were collected and post-fixed overnight in 4% PFA at 4°C, followed by two nights in 30% Sucrose. Tissues were frozen, cut into 40μm sections using a cryotome (Leica) and stained as described previously (Wegleiter et al., 2019) using the same antibodies against mCherry and GFP. Nuclei were counterstained using DAPI (1:1000).

To compare iCOUNT positive and iCOUNT negative embryos, brain sections of E15.5 embryos were stained against mCherry (1:250, Thermo Fisher Scientific), SOX2 (1:250, rat, eBioscience), KI67 (1:250, rat, eBioscience), cleaved Caspase-3 (1:250, BioConcept), and phosphorylated histone 3 (pH3, 1:250, Abcam). FIJI was used for image analysis whereas the SOX2 or KI67 positive area was determined using constant thresholding and divided by the total cortical area (as indicated in Figure S3B), the pH3 positive area was divided by the ventricular area (as indicated in Figure S3B), and the number of Caspase-3 positive cells was counted and extrapolated to cells/mm^3^. Mean values were plotted (±standard deviation, n = 6 cortices derived from 3 embryos).

Adult brain sections of wild-type C57BL/6 (WT), iCOUNT-positive and iCOUNT- negative animals were stained against mCherry (1:250, rabbit, Thermo Fisher Scientific), HOPX (1:250, mouse, Santa Cruz), SOX2 (1:250, rat, Invitrogen), DCX (1:350, guinea pig, Millipore), KI67 (1:250, rat, eBioscience), and cleaved Caspase-3 (1:250, BioConcept). R cells (HOPX+, SOX2+, radial process), dividing NR cells (KI67+, HOPX+, SOX2+, no radial process), and apoptotic cells (Caspase-3+) were manually counted and extrapolated to cells/mm^3^. The amount of newborn neurons was compared by dividing the DCX+ area determined by constant thresholding by the total granule cell layer (GCL, outlined in Figure S5C). Mean values were plotted (±standard deviation, n = 12 DGs derived from 6 iCOUNT animals (divided into expressing and non-expressing) and 12 DGs derived from 6 WT animals.

To quantify the proliferation of WT and iCOUNT mESCs, cells were plated on Lab Tek II chambered coverglasses (Thermo Fisher Scientific) coated with Poly-L-ornithine (10 μg/ml, Sigma-Aldrich) and Laminin (5 μg/ml, Sigma-Aldrich) 24h prior to the experiment. Then, cells were incubated with EdU containing media (10 μM, Thermo Fisher Scientific) for 1h. Cells were fixed with PFA (4%) for 5min, labeled for EdU using the Click-iT EdU AF647 Proliferation Kit (Thermo Fisher Scientific), and nuclei were counterstained with DAPI (1:1000) prior to imaging. Pictures were analyzed using FIJI and mean EdU+ cells as percentage of all cells was plotted (±standard deviation, n = 6 independent chambers).

To analyze DNA damage and apoptotic cell death in WT and iCOUNT mESCs, cells were plated on Poly-L-ornithine and Laminin coated chambers 24h prior to fixation with PFA (4%) for 5min. Antibodies detecting phosphorylated H2AX (1:250, Biolegend) and cleaved Caspase-3 (1:250, BioConcept) were used and cells were counterstained with DAPI (1:1000). To test for Caspase-3 and H2AX antibody specificity, cells were treated for 1h with hydroxyurea (10mM; Sigma Aldrich). Images were analyzed using FIJI. The area of H2AX was determined using constant thresholding and the percentage of H2AX area of DAPI area was calculated. The number of Caspase-3 positive cells was divided by the number of total cells. Mean (±standard deviation, n = 5 independent chambers) was plotted.

#### Multiplexed immunostaining

To assess the cell division history of adult neural stem cells, adult H3.1-iCOUNTxGli1:CreER^T2^ mice were injected with TAM (180mg/kg) three times constitutively (with one day intervals) and animals were sacrificed by perfusion two weeks after the first TAM injection (n = 3 animals). Brains were sectioned coronally into 40μm sections that were mounted on poly-D-lysine (PDL, Sigma-Aldrich) pre-treated glass-bottomed 24 well-plates (Cellvis). Sections were stained using iterative indirect immunofluorescence imaging (4i) as described previously (Gut et al., 2018), adapted for fresh-frozen tissue. Tissues were stained for mCherry (1:250, rabbit, Thermo Fisher Scientific), GFP (1:250, goat, Rockland), HOPX (1:250, mouse, Santa Cruz), GFAP (1:500, chicken, Novus), SOX2 (1:250, rat, Invitrogen), S100β (1:500, rabbit, Abcam), DCX (1:350, goat, Santa Cruz), KI67 (1:250, rat, eBioscience), OLIG2 (1:500, rabbit, Millipore), IBA1 (1:500, rabbit, Wako) and NEUN (1:250, mouse, Millipore). During imaging, fluorescent cross-linking was prevented by using phosphate buffer containing N-Acetyl-Cysteine (0.7M, Sigma-Aldrich) at pH 7.4. After imaging, antibodies were eluted using a buffer (pH 2.5) containing L-Glycine (0.5M, Sigma-Aldrich), Urea (3M, Sigma-Aldrich), Guanidinium Chloride (3M, Sigma-Aldrich) and TCEP-HCL (0.07M, Sigma-Aldrich). Tissues were washed and next round of antibodies was applied using a standard staining protocol (Wegleiter et al., 2019). Nuclei were counterstained with DAPI (1:1000) in every round of staining to allow image alignment for image analysis.

To analyze H2B-GFP fluorescence intensity in the brain of loaded (no chase; n = 3 animals) or loaded and chased animals (chase; n = 3 animals), brains were sectioned coronally into 40μm sections and DG containing sections were mounted on PDL pre-treated glass-bottomed 24 well-plates. Sections were stained for HOPX, SOX2, S100β, DCX, KI67, IBA1 and NEUN and nuclei were counterstained with DAPI using the 4i technology as described above.

To stain different cell types in human organoids, day 32 old organoids were injected with Cre expressing plasmids (1 μg/μl) and electroporated using the AMAXA nucleofector device. 7 days post Cre expressing plasmid injection, organoids were fixed using 4% PFA for 30min, transferred to 30% sucrose solution, embedded in OCT compound and frozen using liquid nitrogen. Organoids were sectioned into 40 μm slices using a cryostat, collected in PBS and then mounted on PDL coated glass-bottomed 24 well-plates as described above. 4i was performed using antibodies against SOX2 (1:100, mouse, R&D Systems), KI67 (1:100, mouse, BD-PharMingen), TBR2 (1:100, rabbit, Abcam), PAX6 (1:100, rabbit, Biolegend), DCX (1:300, goat, Santa Cruz), CTIP2 (1:100, rat, Abcam) and TUJ1 (1:150, mouse, Biolegend). Nuclei were counterstained using DAPI (1:1000).

#### Fixed cell image analysis

Images were analyzed using Fiji. 4i images of different rounds of staining were aligned by DAPI signal and assembled using Fiji. Whole nuclear fluorescence (integrated density) was measured and background was subtracted for every cell independently. Red fluorescence was normalized by the average of 10 darkest (set to 0%) and 10 brightest “red-only” nuclei (set to 100%) in the analyzed tissue. Green fluorescence was normalized by the brightest value (set to 100%) in the analyzed tissue. Percentage green fluorescence was calculated by dividing the green fluorescence by total fluorescence (sum of green and red fluorescence). Percentage of green fluorescence was calculated for every cell and plotted grouped by different cell type or localization as indicated. Predicted division number was calculated depending on the percentage green fluorescence ranging from 0 to 50% (0-1 division), 50% to 75% (1-2 divisions), 75% to 87.5% (2-3 divisions) 87.5% to 93.75% (3-4 divisions) and more than 93.75 (4+ divisions). Values of single cells (±standard deviation, n ≥ 28 cells (Figure 3D), n ≥ 21 cells (Figure 4B), n ≥ 90 cells (Figure 4E), n ≥ 10 cells (Figure 5E), n ≥ 15 cells (Figure 6E) were plotted derived from ≥ 3 mice, embryos or organoids.

To assess the intensity of H2B-GFP loaded cells, mean fluorescence was measured and background was subtracted. H2B-GFP signal was measured in R cells (HOPX+, SOX2+ with radial process), NR cells (HOPX+, SOX2+ without radial process), newborn neurons (DCX+) and neurons (DCX- and NEUN+) containing detectable GFP signal.

To assess the symmetric distribution of red and green fluorescence between daughter cells, the ratio of green over red fluorescence was measured on both sides of anaphase cells and values were pairwise plotted (n = 7 cells). To test the effect of staining, the percentage of green fluorescence was measured in fixed mESCs stained against GFP (using 405nm secondary antibodies) and mCherry (using Cy5 secondary antibodies, Jackson Immuno Research). The percentage of green fluorescence from the endogenous signal (GFP/mCherry) was pairwise plotted to the percentage of green fluorescence from stainings (405 nm/Cy5, n = 13 cells).

#### Theory – cell division histories during cortical development

To assess the utility of the iCOUNT system as a quantitative tool to study cell cycle progression, we made use of existing knowledge of the clonal dynamics during mouse cortical neurogenesis to examine the distribution of cell cycle number. To prepare the analysis, we first considered the predicted distribution using the results of quantitative clonal fate studies by Gao and colleagues, based on the MADM reporter system (*12*) (for details, see Methods S1). The iCOUNT system predicts that after n rounds of cell division, the mCherry fluorescence intensity signal is a factor of 1/2^n^ smaller than its original level as it becomes replaced by GFP signal. From the ratio of the fluorescence intensity of the mCherry and GFP, we obtained an estimate of the cell division number. Therefore, defining

*I*_mCherry_ = I^(0)^_mCherry_/2^n^ and I_GFP_ = I^(0)^_GFP_ (1−1/2^n^), we have the ratio

R=IGFPIGFP+ImCherry=11+C(2n−1)

where C = I^(0)^_mCherry_/ I^(0)^_GFP_. If the baseline flourescence signals are equally intense for both reporters, *C* = 1, and n = − ln(1 − *R*) / ln2. Based on this analysis, the predicted distribution of cell cycle number from the iCOUNT data from the E11.5-E19.5 chase and from the E14.5-E16.5 chase was compared with theoretical predictions from a model obtained from the clone fate analysis of Gao et al. Further details on the comparison are described in the Methods S1.

#### FACS analysis of *in vitro* samples

mESCs expressing H3.1-iCOUNT or NUP155-iCOUNT were transfected with Cre, whereas mNSPCs expressing H3.1-iCOUNT were electroporated with Cre, plated on Laminin coated plates, re-plated the next day and 6h later media was exchanged to keep proliferation, induce differentiation or quiescence (as described above). At the time points indicated on the figures, mNSPCs and mESCs were collected using TrypLE (Thermo Fisher Scientific). Cells were stained with the live/dead marker Zombie Violet (Biolegend) and analyzed on an LSR II Fortessa (BD Biosciences). GFP and mCherry expression levels were quantified in singlet-gated live cells using FlowJo (Tree Star).

CFSE analysis in quiescent NSPCs was performed as follows: WT NSPCs were collected and stained with CFSE (5 μM, Thermo Fisher Scientific) according to the manufacturer’s user guide. Short, NSPCs were collected, diluted in 1ml DPBS containing CFSE and incubated for 20min at 37°C protected from light. After adding 5ml of DMEM medium and incubating cells at 37° for 5min, cells were washed, resuspended in quiescence inducing medium and plated on 6 well plates previously coated with Poly-L-ornithine and Laminin. 3 days after labeling and induction of quiescence, the first well was collected for FACS analysis. From day 3-7, medium was changed daily (containing BMP4 and FGF-2) and every day one well was incubated with EdU (10 μM, Thermo Fisher Scientific) for 24h, cells were collected, stained against EdU using the Click-iT EdU AF647 Flow Cytometry Assay Kit (Thermo Fisher Scientific) following the manufacturer’s instructions and analyzed on the LSR II Fortessa. For CFSE quantifications only EdU negative cells were used. To assess the H2B-GFP intensity in quiescent cells, WT mNSPCs were collected and electroporated with pCAG:H2B-EGFP. Cells were plated on Poly-L-ornithine and Laminin coated 6-wells in BMP4, FGF-2, and Dox containing media to induce quiescence and load cells with H2B-GFP. 3 days post induction media was changed to quiescence media without Dox. Each day one well was labeled with EdU and cells were collected and analyzed as described above.

hESCs expressing NUP155-miCOUNT ER^T2^-Cre-ER^T2^ were first treated with TAM (2.5 μM 4-hydroxytamoxifen, Sigma) for two days before electroporation with Flp (pCAG-Flpe, Addgene No 13787). Two days post electroporation, cells were collected using Accutase, stained with Zombie NIR (Biolegend) and analyzed using the LSR II Fortessa. GFP, tdTom and BFP expression levels were quantified in singlet-gated live cells using FCS Express.

Human miCOUNT organoids were injected with Cre expressing plasmids (0.5 μg/μl) together with control plasmid (0.5 μg/μl) or RPL38 OE plasmid (0.5 μg/μl) and electroporated. At 35 days in culture, 3, 5, or 7 days post injection, organoids were dissociated in Accutase (Sigma-Aldrich) for 30min. After dissociation, cells were washed, resuspended in DPBS and FACS analyzed using the LSR II Fortessa (BD Biosciences). GFP and mCherry expression levels were quantified in singlet-gated live cells using FlowJo (Tree Star). For each time point the GFP positive cells were gaited into GFP low (orange) and GFP high (green) cells. The percentage of GFP high cells was then plotted for control and RPL38 OE conditions. N = 3 independent electroporations per condition and time point.

#### FACS analysis of mouse tissues

The nuclei dissociation protocol was similar to the previously described protocol (Jaeger et al., 2018). Briefly, the dentate gyrus was carefully excised and immediately placed into a nuclei isolation medium (0.25M sucrose, 25mM KCl, 5mM MgCl2, 10mM TrisHCl, 100mM dithiothreitol, 0.1% Triton, protease inhibitors). Tissue was Dounce homogenized, allowing for mechanical separation of nuclei from cells. The nucleic acid stain Hoechst 33342 (5 μM, Life Technologies) was included in the media to facilitate visualization of the nuclei for quantification. Samples were washed, resuspended in nuclei storage buffer (0.167M sucrose, 5mM MgCl2, 10mM TrisHCl, 100mM dithiothreitol, protease inhibitors) and filtered. Solutions and samples were kept cold throughout the protocol. For RNA-seq experiments, tools and solutions were made RNase-free and RNase inhibitors were used (Promega, 1:1000 in both isolation and storage buffers). Bone marrow cells were flushed from the tibiae and femurs of mice and red blood cells were lysed using the RBC Lysis Buffer (Biolegend). Hoechst 33342 was added for live cell discrimination. Skin cells were isolated using a modified version of the protocol by Kostic and colleagues (Kostic et al., 2017). Briefly, the dorsal skin of mice was dissected and the connective tissues and fat were removed. The skin was then incubated in PBS without Ca^2+^ and Mg^2+^ (DPBS^–^) with 0.25% Trypsin at 37°C. 2h later, the hair was scrapped off and the skin was triturated using 10 mL pipettes. The cell suspension was filtered successively through 70 and 40 μm cell strainers (Sigma-Aldrich) and washed twice with DPBS^–^ with 3% chelated FBS. Hoechst 33342 was added for live cell discrimination. Dentate gyrus, bone marrow and skin samples were analyzed on an LSR II Fortessa (BD Biosciences). GFP and mCherry expression levels were quantified in singlet-gated live cells using FlowJo (Tree Star).

#### Single-cell and single-nuclei sorting

Tamoxifen (180mg/kg) was injected into pregnant H3.1-iCOUNT x ROSA26:CreER^T2^ mice at E13.5. 38h later, embryonic cortices were dissected and the nuclei were dissociated as described above (see FACS analysis of mouse tissues). Organoids were injected with Cre expressing plasmids (1 μg/μl, as described above) and electroporated using the AMAXA Nucleofector device. 4 and 7 days after electroporation (39 days in culture), cells were dissociated as described before (Camp et al., 2015, Jaeger et al., 2020). Short, cells were dissociated using Accutase (Sigma-Aldrich) supplemented with DNase at 37°C for 25min with gentle mixing every 5min. Accutase was removed by washing and cells were resuspended in DPBS containing EDTA (1mM, Sigma-Aldrich) prior to sorting. Singlet-gated live cells were sorted into 384 well plates containing lysis buffer using a FACSAria III sorter (BD Biosciences). Sorted plates were stored at −80°C until library preparation.

#### Single-cell library preparation and sequencing

Library preparation was performed using a mosquito robot HV genomics (TTP Labtech Ltd) following the Smart-seq2 protocol (Jaeger et al., 2020, Picelli et al., 2014). Briefly, 384 well plates containing sorted single nuclei in lysis buffer were thawed and reverse transcription with Superscript II (Thermo Fisher Scientific) and PCR using KAPA Hifi HotStart ReadyMix (Kapa) were performed with Oligodt, TSO and ISPCR biotinylated primers (QIAGEN; see Table S2). Following RT-PCR, clean up with Agencourt AMPure XP beads (Beckman Coulter) was carried out and sample concentrations were measured using Bioanalyzer (Agilent Technologies) and normalized at a concentration of 0.3 ng/μl. The Nextera XT DNA library prep kit (Illumina) was used for subsequent sample preparation. Samples were subjected to a tagmentation reaction, indexing, and PCR amplified. Libraries were then mixed in 384-sample pools and purified with Agencourt AMPure XP beads. Ready DNA libraries were quality controlled using D1000 Screen Tape Assay (Agilent Technologies). Samples were sequenced at the Functional Genomics Center Zurich on Illumina HiSeq 2500 or HiSeq4000 sequencers with single-end 125bp reads.

#### Single-cell RNA-seq analysis

Single-end 126nt-long reads were adaptor removed and trimmed using cutadapt v1.16 and sickle v1.33 with default parameters. Reads were mapped against the mouse GRCm38.90 primary assembly or the human GRCh38.p13 primary assembly using STAR v2.6.0c (Dobin et al., 2013) in `alignReads` mode. Gene count matrices were quantified at the exon level ignoring multimappers using featurecounts from subread v1.6.2. RNA velocity loom files were generated with velocyto v0.17.17 (La Manno et al., 2018) in `run-smartseq2` mode.

Nuclei from the mouse embryonic cortex were quality checked using scater v1.12.2 (McCarthy et al., 2017). Only cells with 1000-3000 genes detected, less than 5% of mitochondrial reads and more than 50’000 reads were kept. In each dataset (i.e., 384 well-plate), nuclei with more or less than 1.5-fold the median read content per cell were excluded. A total of 552 nuclei passed QC filtering. 17 interneuron nuclei (*Gad2*^*+*^*Gad1*^+^) were filtered out. A total of 535 nuclei were retained for final analysis. Human organoid cells were quality checked using scater v1.12.2 (McCarthy et al., 2017). Only cells with 3000-7000 genes detected, less than 10% of mitochondrial reads and more than 50’000 reads were kept. In each dataset (i.e., 384 well-plate), cells with more or less than 1.5-fold the median read content per cell were excluded. A total of 641 cells passed QC filtering. 207 RSPO cells (*RSPO2*^*+*^) were filtered out. A total of 434 cells were retained for final analysis. Seurat v3.1.1 was used to normalize read counts, regress out the library size and mitochondrial reads proportion per cell, find variable features with the `vst` method, integrate multiple plates as in (Stuart et al., 2019), dimensionality reduce the data, cluster cells and test for differentially expressed genes. Differential gene expression between orange and green NSPCs was tested only for genes detected in at least 20% of the cells in either of the populations. Logistic regression fit with batch as latent variable (null model) and cell type as predictor were run using Seurat’s `FindMarkers`. Genes with p value below 0.05 were deemed significant. Seurat v3.1.1 was used to generate t-distributed stochastic neighbor embedding (t-SNE) plots, cluster cells and test for differentially expressed genes (DEGs) using a Wilcoxon Rank Sum test. For pseudotime calculation, independent component analysis was computed with fastICA package v1.2.2. Data analysis was carried out in R v4.0.1. Cytoscape v3.8.2 was used to visualize and analyze the gene networks among DEGs (Shannon et al., 2003). Gene Ontology (GO) analysis were carried out using the StringApp plugin to identify the key Biological Processes (BP) represented in DEGs upregulated in orange or green human organoid NSPCs or neurons. Selected GO pathways were plotted by the -log(10) FDR and the size of the circle represents number of upregulated genes per pathway. Shading of nodes in the STRING network represents the gene’s fold change (average logFC). Functional categories found in both human organoid cells and mouse embryonic cortex nuclei were highlighted.

#### Bulk RNA-seq

WT and iCOUNT mESCs were collected using TrypLE (Thermo Fisher Scientific), washed and resuspended in DPBS containing RNase inhibitor (Promega, 1:1000). RNA extraction was performed using the PureLink RNA Mini Kit (Thermo Fisher Scientific) according to the manufacturer’s protocol. RNA concentration was measured by NanoDrop (Thermo Fisher Scientific) and RNA quality was assessed using TapeStation analysis (Agilent Technologies). PolyA selection was performed using oligo-dT beads (TruSeq RNA Library Prep Kit, Illumina) and libraries were prepared (TruSeq Stranded mRNA, Illumina). Samples were sequenced at the Functional Genomics Center Zurich on a Illumina NovaSeq 6000 with single-end 150bp reads.

### Quantification and statistical analysis

All statistical analyses were done using Prism 8 (GraphPad). First, data was analyzed using a D’Agostino & Pearson normality test. If data was normally distributed, an ordinary One-way ANOVA was performed followed by Tukey’s multiple comparisons (Figures 1E, 2H, 5E, 6E, and S5C). If not, a Kruskal-Wallis test was performed followed by Dunn’s multiple comparisons (Figures 3C, 4B, and S5E). To compare only two groups, an unpaired t-Test was used (Figures 7L, S1C, S1D, and S3B), except when data was not normally distributed, then a two-tailed Mann-Whitney test was used (Figures 4E and S5D). A nested t-Test was used in Figure 7J. To analyze changes in fluorescent intensity in imaged quiescent NSPCs, a repeated-measures one-way ANOVA test was used (Figures S2F and S2G). To investigate the symmetric distribution of histones after division, a paired two-tailed t-Test was used (Figure S1B,S3C, S3D, and S6B). To compare the distribution of R, NR and neurons from iCOUNT and *in vivo* imaging, a Kolmogorov-Smirnov test was used (Figure 5F). No samples were measured repeatedly. Figures 1D, 1E, and S1C as well as S6A and S6B are different quantifications derived from the same measurements. For all experiments, animals were randomly selected and experimenters were blinded for analysis whenever possible.

## Data Availability

All data are available from the authors at request. scRNA-seq and bulk RNA-seq data are available at GEO:GSE167375.

## Supplemental information

Document S1. Figures S1–S7

Table S1. Table of genes differentially regulated in green and orange NSPCs and neurons derived from human brain organoids and mouse developing cortex, related to Figure 7

Table S2. Table of oligonucleotides used in this study, related to STAR Methods

Methods S1. Supplemental information supporting the computational modeling used in Figure 4, related to Figure 4

Document S2. Article plus supplemental information
