# Supplementary material for: Visualization of individual cell division history in complex tissues using iCOUNT
Source: Cell Stem Cell. 2021 Nov 4;28(11):2020–2034.e12. doi: 10.1016/j.stem.2021.08.012 (PMC8577829; doi:10.1016/j.stem.2021.08.012)
Supplement: Document S1. Figures S1–S7 [file mmc1.pdf]

Cell Stem Cell, Volume *28*

Supplemental Information

Visualization of individual cell division history in complex tissues using iCOUNT

Annina Denoth-Lippuner, Baptiste N. Jaeger, Tong Liang, Lars N. Royall, Stefanie E. Chie, Kilian Buthey, Diana Machado, Vladislav I. Korobeynyk, Merit Kruse, Clara M. Munz, Alexander Gerbaulet, Benjamin D. Simons, and Sebastian Jessberger

Supplemental Information for

Visualization of individual cell division history in complex tissues using iCOUNT

Annina Denoth-Lippuner, Baptiste N. Jaeger, Tong Liang, Lars N. Royall, Stefanie E. Chie, Kilian Buthey, Diana Machado, Vladislav I. Korobeynyk, Merit Kruse, Clara M. Munz, Alexander Gerbaulet, Benjamin D. Simons, Sebastian Jessberger

Supplemental Figures

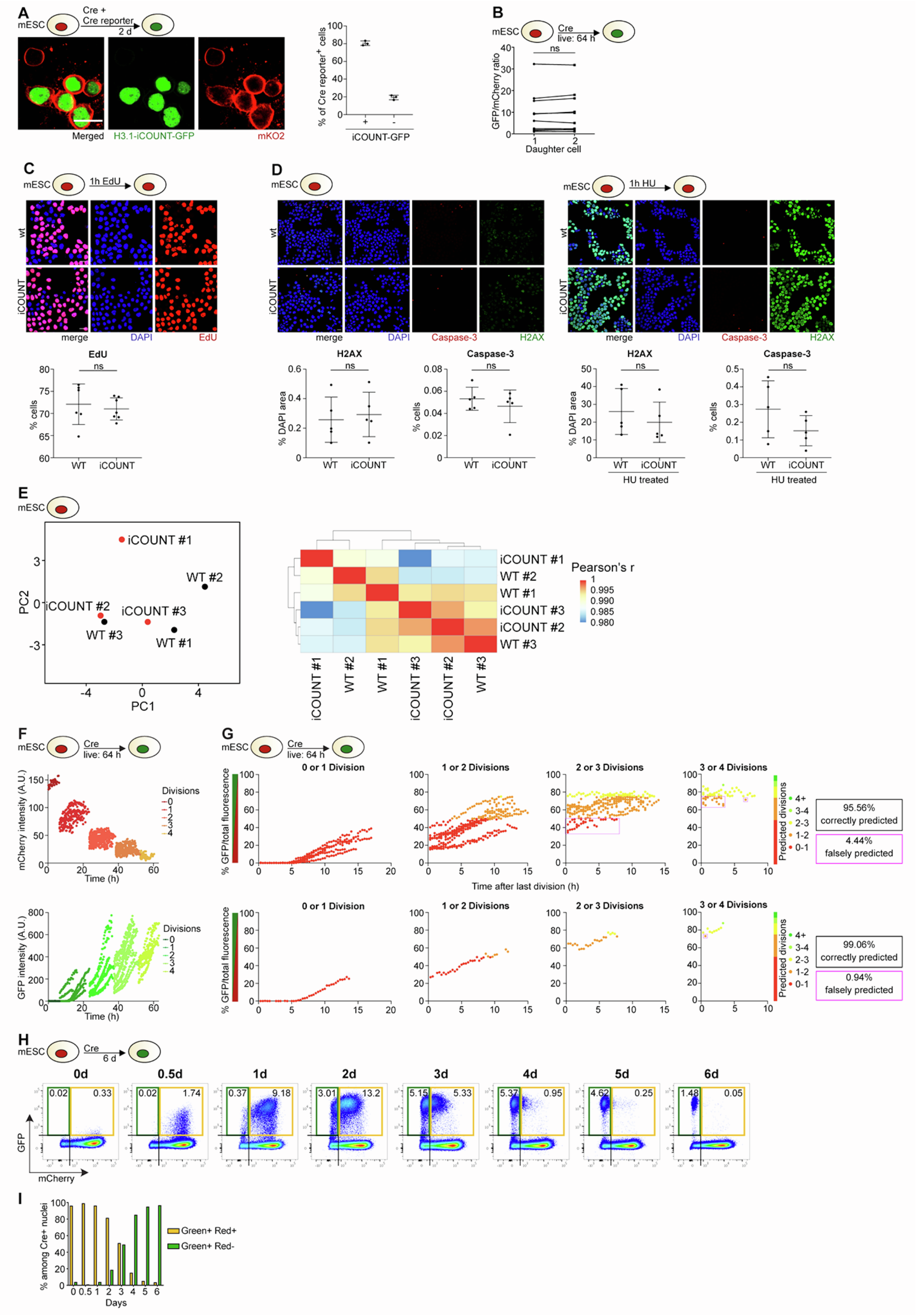

Figure S1: iCOUNT reports cell division events – Related to Figure 1.

(A) Images of GFP-tagged (green) histones in iCOUNT mouse ESCs 2d after co-transfection with Cre and a Cre-reporter plasmid (lox-STOP-lox-mKO2). Left panel shows merged signal. Quantifications show the percentage of Cre-reporter expressing cells that reveal detectable green fluorescence. (B) Quantifications show ratios of green over red fluorescent intensities of sister cell pairs. (C) Images of WT and iCOUNT mouse ESCs treated for 1h with 5-Ethynyl-2'-deoxyuridine (EdU). Quantifications show percentage of EdU positive cells. (D) Images of WT and iCOUNT mouse ESCs stained against Caspase-3 and H2AX with (right panel) or without (left panel) 45 min pre-treatment with hydroxyurea (HU). Quantifications show the H2AX positive area as percentage of DAPI area and the Caspase-3 positive cells as percentage of total cells. (E) Principal component analysis of expressed genes determined using RNA-seq shows homogenous distribution of WT and iCOUNT mouse ESCs. Hierarchical clustering reveals the similarity of WT or iCOUNT cells. (F) Measured changes in fluorescence intensities for red (mCherry) and green (GFP) histones of mESCs imaged over 64h depicting single measurements of single cells (related to Figure 1D). (G) Live cell imaging of iCOUNT mouse ESCs for 64h confirms >95% correct prediction of cell divisions based on fluorescent intensities. Shown are measured data points and their predicted division number for individual observed division bins (upper panel) and one example cell (lower panel; falsely predicted measurements are highlighted in purple). (H) FACS analyses show relative shift from red/green to green fluorescent intensities in mouse ESCs in the course of 6d after Cre-mediated recombination. (I) Quantifications of the boxed areas show the gradual change of iCOUNT colour. Nuclei were counterstained with DAPI (blue). N = 3 biological replicates (A and E); $n \geq 5$ technical replicates (C-D); n = 10 cell pairs

after division (B); N = 4 starting cells and n ≥ 4 daughter cells for each further cell division (F-G). ns, not significant. Images were stitched. Scale bar represents 20μm.

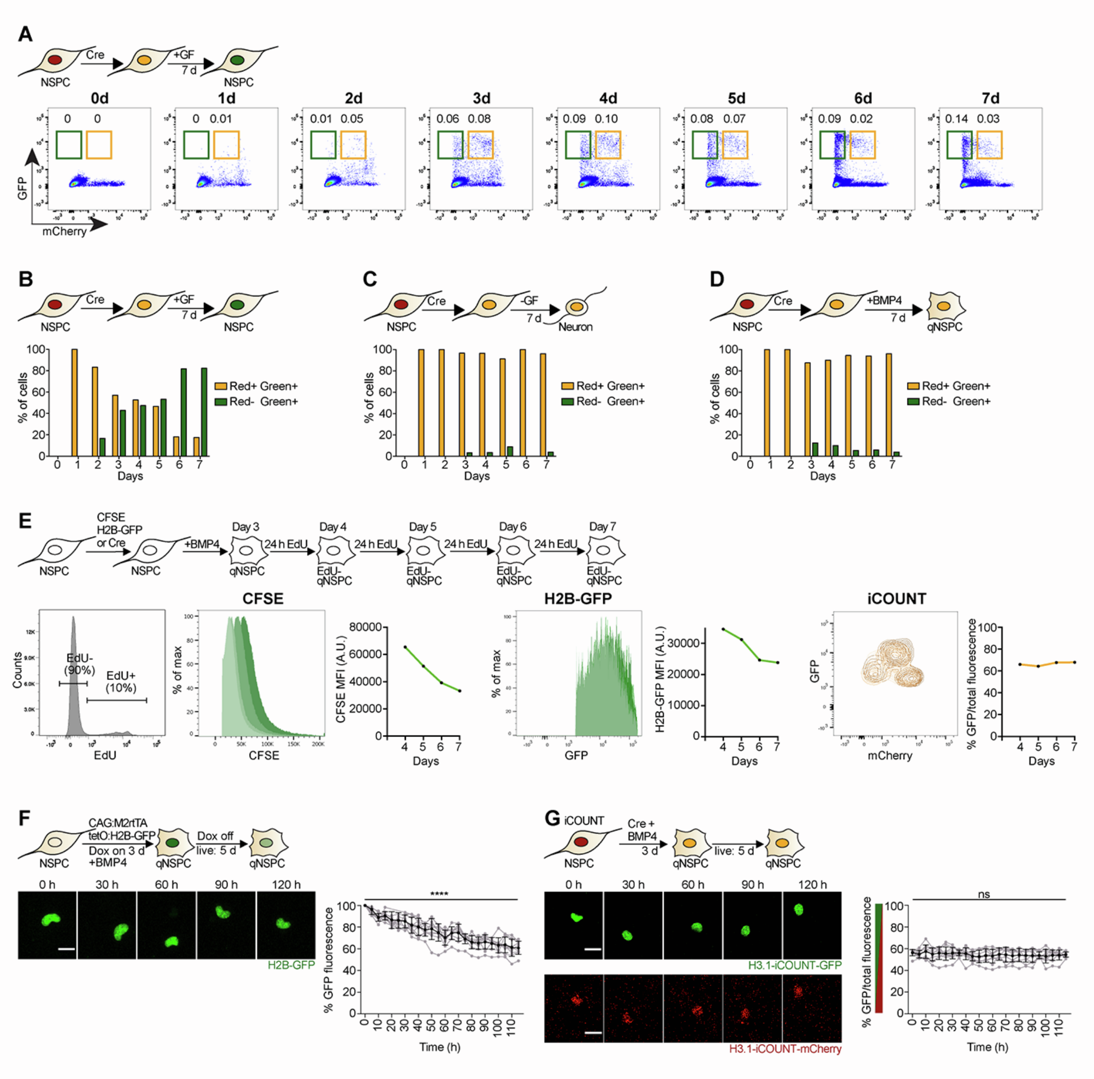

Figure S2: iCOUNT colour is more stable in non-dividing cells compared to CFSE or overexpressed H2B-GFP – Related to Figure 2.

(A) FACS analyses show relative shift from red/green to green fluorescent intensities in mouse NSPCs in the course of 7d after Cre-mediated recombination when exposed to growth factors (GF). (B) Quantification of the boxed areas. (C) In contrast, differentiation by withdrawal of GF (-GF) prevents a fluorescence shift throughout 7d. (D) Inducing quiescence with BMP4 for 7d also prevents a fluorescence shift. (E)

Daily FACS analyses starting 3d after addition of BMP4 to induce quiescence shows decreased fluorescence intensity of CFSE and H2B-GFP but stability of the green/red ratio in iCOUNT cells. Prior to FACS analysis cells are treated with EdU for 24h to permit only truly quiescent cells for the analysis. (F) Live imaging of quiescent NSPCs starting 3d after addition of BMP4 and loading cells with H2B-GFP. Quantifications show the decrease in H2B-GFP fluorescence and images are shown of a representative cell every 30h. (G) Live imaging of quiescent NSPCs starting 3d after addition of Cre and BPM4. Quantifications show the stable fluorescent iCOUNT ratio over the time course of 120h and images are shown of a representative cell every 30h. N = 10 cells (F-G). ****$p < 0.0001$; ns, not significant. Images were stitched. Scale bar represents 20µm.

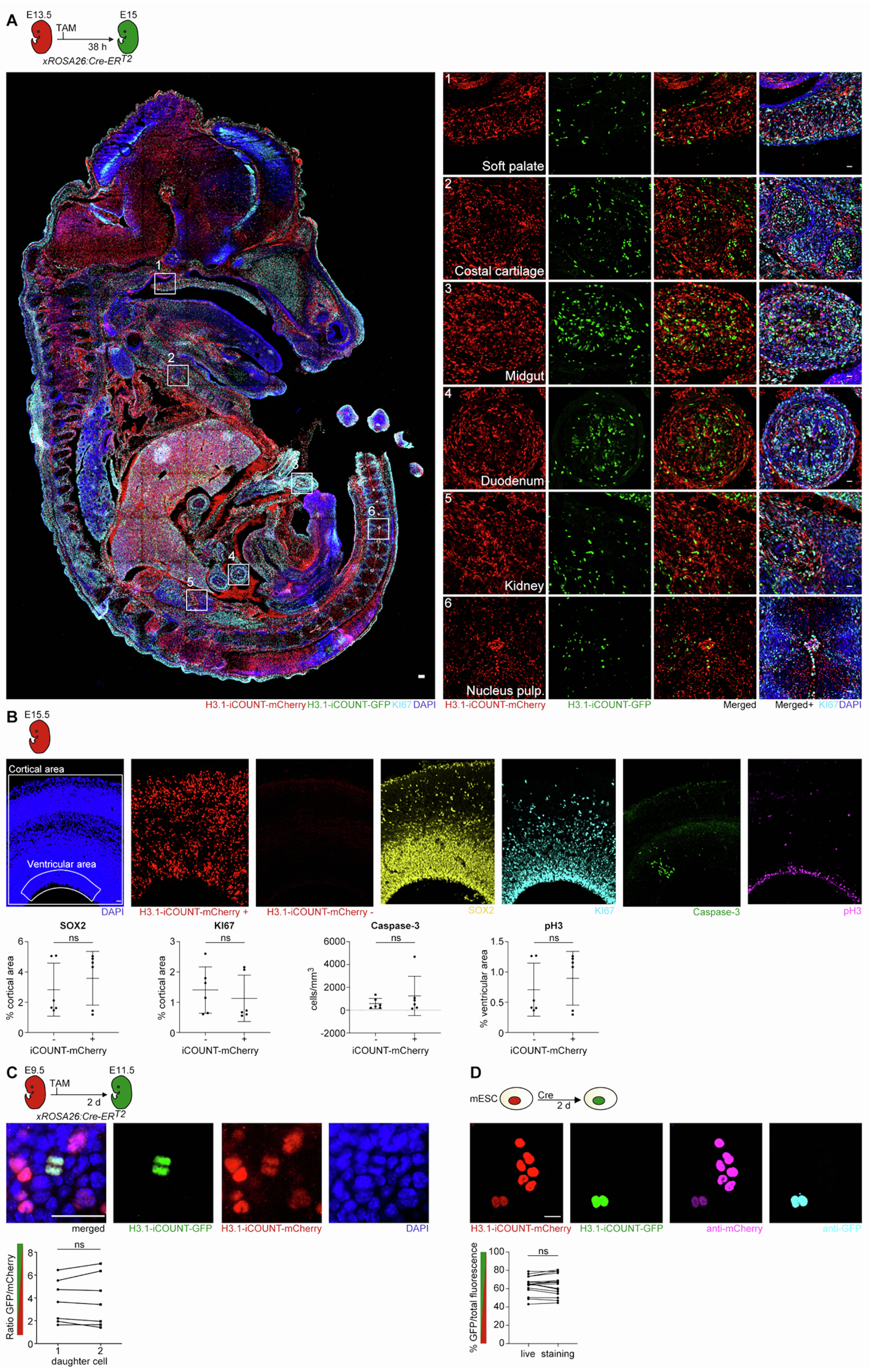

Figure S3: iCOUNT reports cell division history in embryonic tissues – Related to Figure 3.

(A) Overview of iCOUNT mouse at E15 (recombination using TAM at E13.5). Right panels show mCherry (red), GFP (green), KI67 (light blue) signal in tissues indicated. (B) Representative images of sections of E15.5 cortices stained against mCherry (red), SOX2 (yellow), KI67 (turquoise), Caspase-3 (green), and phosphorylated histone 3 (pH3; magenta). Quantifications show no differences between iCOUNT expressing and non-expressing embryos for all markers. Boxed areas in the DAPI image indicate the analysed areas. (C) Symmetric segregation of mCherry- (red) and GFP-tagged (green) histones in the developing mouse cortex in dividing progenitors. Right panel shows quantification of green over red fluorescent intensities for sister pairs. (D) Amplification of endogenous iCOUNT signal in mESCs of mCherry (red) and GFP (green) using antibodies against mCherry (pink) and GFP (turquoise) maintains relative fluorescence ratios. Quantifications of analysed cells are shown in right panel. Nuclei were counterstained with DAPI (blue). N = 6 cortices derived from 3 embryos (B); n = 7 cell pairs after division (C); n = 13 cells (D). ns, not significant. Images were stitched. Scale bars represent 100µm in A (left panel) and 20µm in A (right panels), B-D.

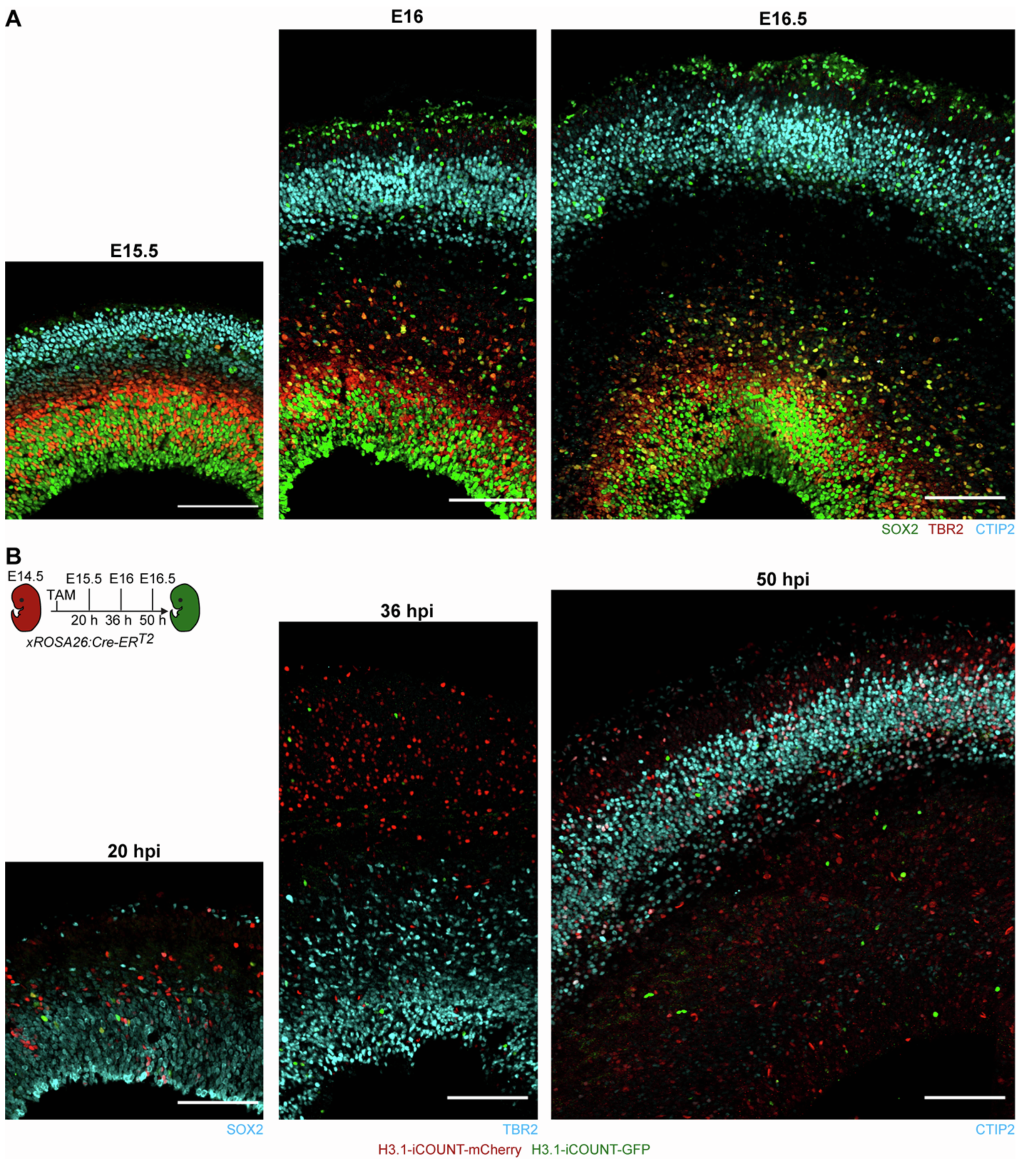

Figure S4: Recombined iCOUNT cells in different areas of the developing cortex – Related to Figure 4.

(A) Images of the developing mouse cortex analysed at E15.5, E16 and E16.5 stained with SOX2 (green), TBR2 (red) and CTIP2 (light blue) depicting different layers. (B) Representative images showing iCOUNT-labelled cells containing H3.1-mCherry (red), H3.1-GFP (green) and SOX2, TBR2 or CTIP2 (light blue) in the developing cortex of iCOUNT mice induced at E14.5 and analysed at indicated time points. Images were stitched. Scale bars represent 100µm.

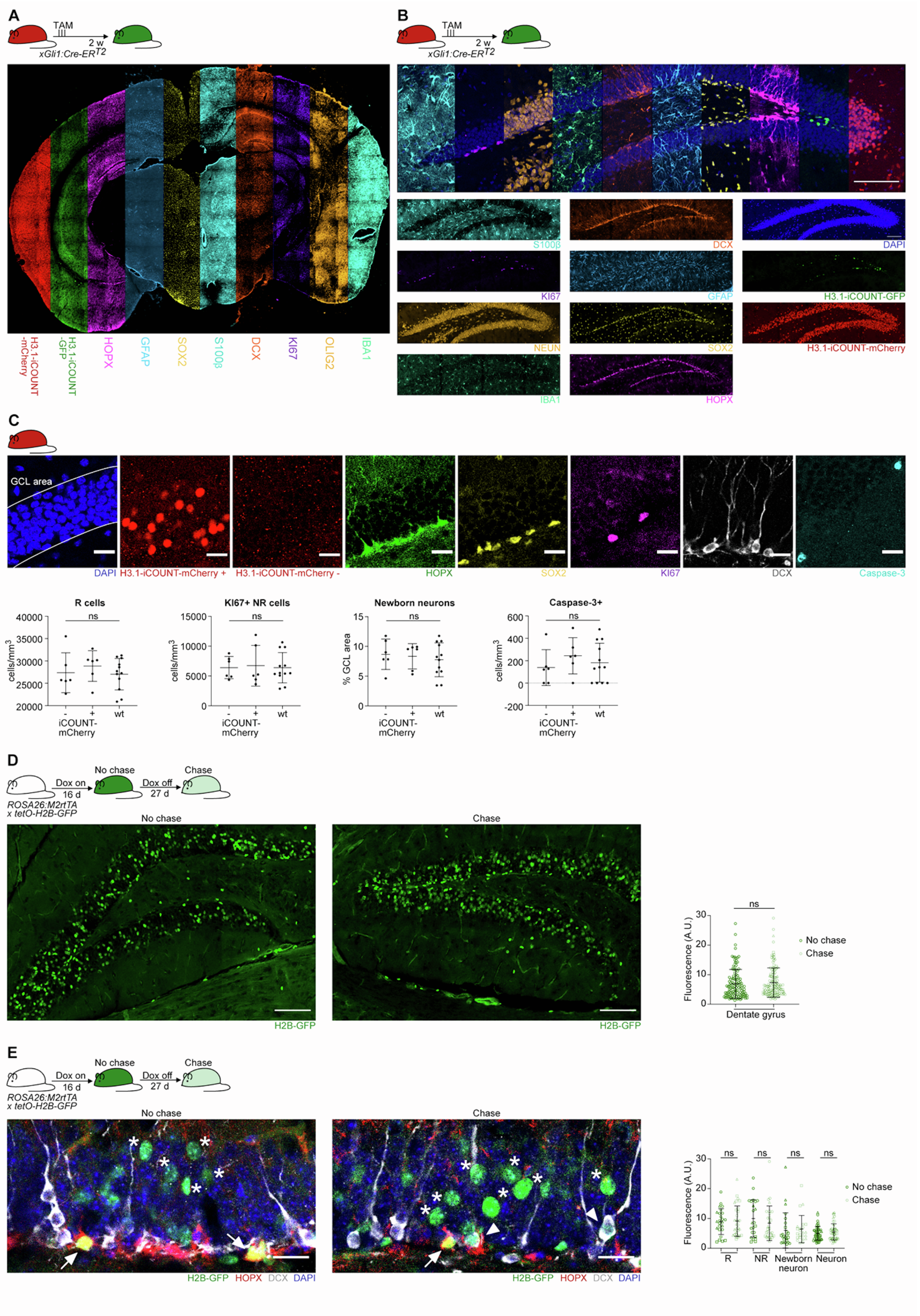

Figure S5: iCOUNT and H2B-GFP signal in adult mouse tissues – Related to Figure 5.

(A) Overview of an adult iCOUNT mouse brain with expression of the tagged H3.1 and analysed using 4i technology with the markers indicated. (B) High power view of the mouse hippocampus and analysed using 4i technology with the markers indicated. (C) Representative images of the DG of WT animals, iCOUNT expressing, and non-expressing animals stained with antibodies against mCherry (red), HOPX (green), SOX2 (yellow), KI67 (magenta), DCX (grey), and Caspase-3 (turquoise). Quantifications show no differences in number of R cells (HOPX+, SOX2+, radial process), proliferating NR cells (KI67+, HOPX+, SOX2+, no radial process), newborn neurons (DCX+), and apoptotic cells (Caspase-3+). (D) Images of the hippocampus of mice loaded for 16d with H2B-GFP and analysed immediately (left panel) or after 27d of chase (right panel). Quantifications show the broad distribution of H2B-GFP fluorescence among different cells in the dentate gyrus both with and without chase. (D) Zoom in pictures of representative areas showing different cell types (R cells with arrows, newborn neurons with arrow heads and neurons with stars). Quantifications show the H2B-GFP fluorescence of R cells, NR cells, newborn neurons and neurons before and after the chase. Nuclei were counterstained with DAPI (blue). N $\geq$ 6 DGs derived from $\geq$ 3 animals (C); n $\geq$ 125 cells (D) and n $\geq$ 23 cells (E) derived from 3 animals. ns, not significant. Images were stitched. Scale bars represent 100µm in A-B and D and 20µm in C and E.

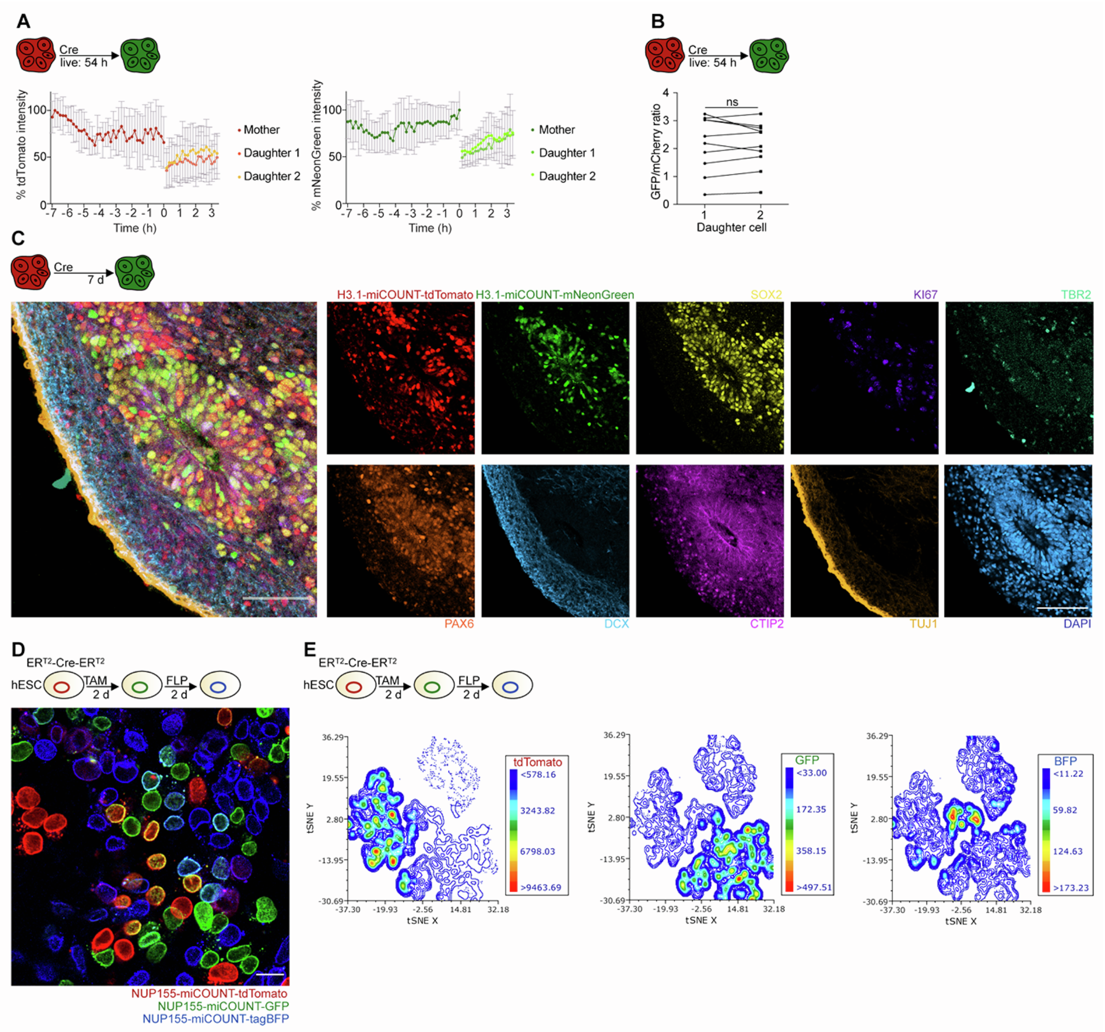

Figure S6: iCOUNT and miCOUNT reports cell division events in human cells – Related to Figure 6.

(A) Quantification of measured changes in fluorescence intensities for red and green histones of iCOUNT-targeted cells in the organoid during live imaging aligned by the time of division (mean ± SD). (B) Symmetric segregation of tdTomato- (red) and mNeonGreen-tagged (green) histones in human organoids upon cell division in sister pairs. Graph shows quantification of green over red fluorescent intensities for sister pairs. (C) 4i-based phenotyping of iCOUNT-targeted cells in human ESC-derived forebrain organoids using a panel of protein markers as indicated. (D) NUP155-miCOUNT expressing human ESCs 2d after TAM and 2d after FLIP-based

recombination. Note the presence of tdTomato-tagged (red), GFP-tagged (green), and tagBFP-tagged (blue) NUP155 and their combinations in human ESCs. (E) FACS analyses confirm the presence of red, green, blue fluorescent intensities in human NUP155-miCOUNT expressing ESCs, depicted using a tSNE plot of fluorescence values. N = 11 cells (A); n = 9 cell pairs after division (B). ns, not significant. Images were stitched. Scale bars represent 100µm in C and 20µm in D.

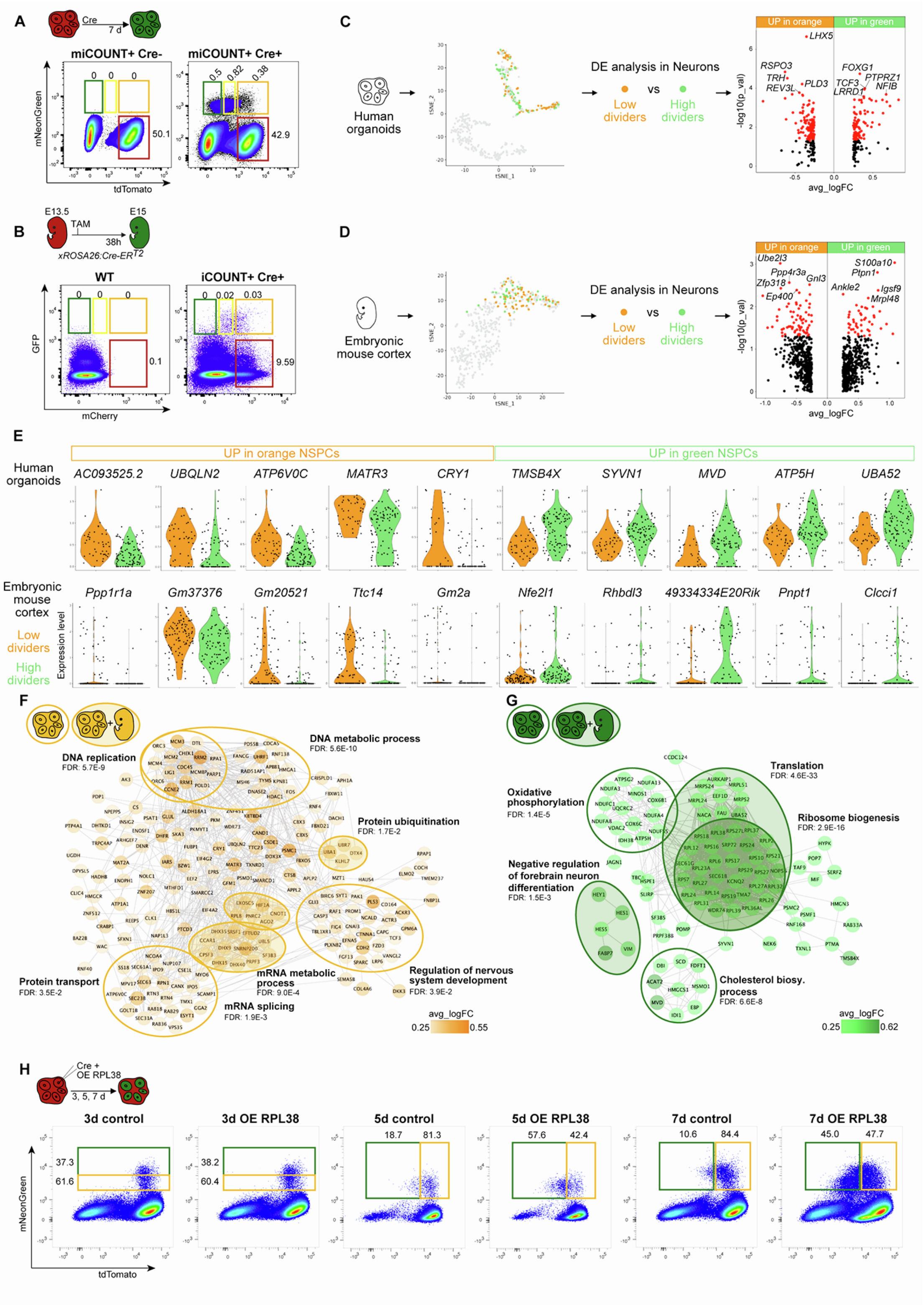

Figure S7: Molecular consequences of previous cell divisions in mouse and human NSPCs and neurons – Related to Figure 7.

(A) FACS analyses of cells with distinct red/green histone ratios in human forebrain organoids 7d after induction of Cre. Shown are FACS plots of cells without (left) and

with induction of Cre (right panel). (B) FACS analyses of cells derived from developing mouse cortex at the time points indicated. Shown are FACS plots of WT control animals (left) and iCOUNT mice 38h after induction of Cre (right panel). Boxed areas in different colours depict gating strategies to collect single cells of different iCOUNT colours used for scRNA-seq. (C) Volcano plot showing differentially expressed genes (p-value < 0.05 in red) between orange and green neurons derived from human brain organoids and (D) from the mouse developing cortex. (E) Violin plots showing the top five genes upregulated in orange (left) and green (right) NSPCs derived from human organoids (upper panel) or mouse cortex (lower panel). (F) GO enrichment analyses of cells with low (orange) cell division history within the NSPC cluster of human organoids. GO terms shown in shaded circles were shared between human organoids and developing mouse cortex. Different shades of orange represent different fold changes (avg_logFC). (F) GO enrichment analyses of cells with high (green) cell division history within the NSPC cluster of human organoids. GO terms shown in shaded circles were shared between human organoids and developing mouse cortex. Different shades of green represent different fold changes (avg_logFC). (H) Representative FACS plots of cells derived from human miCOUNT organoids 3, 5, and 7 days after injections with Cre and either a control or RPL38 overexpressing plasmid. Boxed areas were used for quantifications in Figure 7L.
