# Supplementary material for: Visualization of individual cell division history in complex tissues using iCOUNT
Source: Cell Stem Cell. 2021 Nov 4;28(11):2020–2034.e12. doi: 10.1016/j.stem.2021.08.012 (PMC8577829; doi:10.1016/j.stem.2021.08.012)
Supplement: Methods S1. Supplemental information supporting the computational modeling used in Figure 4, related to Figure 4 [file mmc4.pdf]

Methods S1: Computational Modeling - Related to Figure 4.

To illustrate the utility of the iCOUNT system as a quantitative tool to study cell cycle progression, we made use of prior knowledge of clonal dynamics during mouse cortical neurogenesis to examine the distribution of cell cycle number. To prepare the analysis, we first considered the predicted distribution using the results of quantitative clone fate studies by Gao and colleagues, based on the MADM reporter system (Gao et al. 2014).

Predicted cell cycle number distribution: Based on these findings, cortical development comprises three distinct phases. In the first "amplification" phase (ca. E9-E12), radial glial progenitors (RGPs) move step-wise through a sequence of n_s symmetric divisions before abrupt entry into the second neurogenic phase, i.e. for an RGP at "generation" g in the amplification phase,

$$\mathrm{R}_g^{\mathrm{A}} \mapsto \begin{cases} \mathrm{R}_{g+1}^{\mathrm{A}} + \mathrm{R}_{g+1}^{\mathrm{A}} & \text{if } 1 \leq g < n_s \\ \mathrm{R}_1^{\mathrm{N}} + \mathrm{R}_1^{\mathrm{N}} & \text{if } g = n_s \end{cases}$$

The length of the amplification phase is not known precisely, and indeed may vary across different RGPs; but the MADM clone data suggests $n_s \sim 5$.

In the neurogenic phase (ca. E12-E16), RGPs follow a pattern of invariant asymmetric fate, with each cell division giving rise to one RGP and either a neuron (with probability q) or an intermediate progenitor, P (with probability $1 - q$), i.e. for an RGP at generation g in the neurogenic phase,

$$\mathrm{R}_g^{\mathrm{N}} \mapsto \begin{cases} \mathrm{R}_{g+1}^{\mathrm{N}} + \mathrm{N} & \mathrm{Pr.}\, q \\ \mathrm{R}_{g+1}^{\mathrm{N}} + \mathrm{P} & \mathrm{Pr.}\, 1-q \end{cases} \qquad \text{if } 1 \leq g < n_p$$

Once again, the precise range of the asymmetric phase is uncertain, but an estimate of $n_p \sim 5$ is consistent with the clone data.

Finally, while the fate behaviour of intermediate progenitors is also difficult to resolve precisely, with their output limited to at most three neurons, we can capture their dynamics by assuming that they choose between asymmetric division (with probability $p \ll 1$) and terminal division (with probability $1-p$), giving rise to neuronal progenies,

$$\mathrm{P} \mapsto \begin{cases} \mathrm{P}+\mathrm{N} & \mathrm{Pr.}\, p \\ \mathrm{N}+\mathrm{N} & \mathrm{Pr.}\, 1-p \end{cases}$$

Therefore, in the neurogenic phase, for each round of asymmetric division, an RGP generates an average output of

$$\bar{n} = q + (1-q)\sum_{n=1}^{\infty}(n+1)(1-p)p^{n-1} = q + (1-q)\frac{(2-p)}{(1-p)}$$

From MADM tracings from the E12 time-point, analysis of sister clones suggests that $q \simeq 0.3$ and, from the measured average neurogenic output of a single RGP division $\bar{n} \simeq 1.87$, it follows that $p \simeq 0.2$.

Finally, after n_p rounds of asymmetric division, a fraction of RGPs then progress into a gliogenic phase (at around E16), where they give rise to astrocytes and oligodendrocytes. Since we are interested here in the neuronal outputs of RGPs, it is not necessary to consider further the fate behaviour within this phase.

Cell cycle number distribution: Based on this dynamics, as a starting point, we first considered the expected distribution of cell cycle number of neuronal outputs

based on the induction of a RGP that is at some generation g within the neurogenic phase. Defining Q_n as the probability of finding a neuron that has experienced precisely n rounds of division following induction of its RGP ancestor, we have that

$$Q_n = \frac{1}{1-2^{-(n_p-g)}} \sum_{m=1}^{\min(n_p-g,n)} \sum_{r=0}^{\infty} \frac{1}{2^m} d_r \delta_{n,m+r}$$

where

$$d_n = \frac{(1-p)}{(2-p-q)} \Big[q\delta_{n,0} + (1-q)[2(1-p)p^{n-1} + p^n](1-\delta_{n,0})\Big]$$

Here, the first term under the sum represents the one-half probability that, following the RGP division, the lineage follows a RGP fate rather than a N/P fate. The second term represents the probability d_r that, once a N/P fate is adopted, there are r rounds of division before terminal differentiation. Note that here, since the progenitor differentiates through terminal division, there is a factor of 2 that enters the second component of the sum in the definition of d_n.

In practice, if the RGP is labelled early in the neurogenic phase, the majority of neurons will derive from a generation $n \ll n_p$. In this case, we can make the approximation $n_p \to \infty$, whereupon

$$Q_n \simeq \sum_{m=1}^{n} \frac{1}{2^m} d_{n-m} = \frac{(1-p)}{(2-p-q)} \begin{cases} q/2 & n=1 \\ \frac{(1-q)(2-p)p^n - 2^{-n}p(4-2p+3q)}{p(1-2p)} & n>1 \end{cases}$$

In this limit, the average number of cell divisions experienced before neuron pro-

duction is given by

$$\langle n\rangle = \sum_{n=1}^{\infty} nQ_n = 3 + \frac{1}{1-p} - \frac{(2-p)}{(2-p-q)}$$

Then, for a RGP labelled at generation g during the amplification phase, a total of 2^{g-n_s+1} RGPs will enter into the neurogenic phase. So, if all labelled RGPs were synchronized, the distribution of cell cycle number of neurons gener-ated from such RGPs would be simply Q_{n+g-n_s}. However, in practice, RGPs are not synchronized in their progression through the amplification phase, but instead belong to a distribution of generation number which, empirically, fits well with the following dependence (Gao et al. 2014),

$$F_g(t) \simeq k(t)\delta_{g,0} + (1-k(t))\frac{1}{C(t)}2^{g-a(t)}e^{-2^{g-a(t)}}(1-\delta_{g,0})$$

where $a(t)$ scales linearly with time, $k(t)$ denotes the probability that, at the time of induction, the RGP is already in the neurogenic phase, and $C(t) = \sum_{g=1}^{n_s} 2^{g-a(t)}e^{-2^{g-a(t)}}$ denotes the total normalization. At the E12 induction time, measurements based on the MADM system suggest a figure of $k(t) \simeq 0.72$, i.e. some 72% of RGPs have already entered into the neurogenic phase.

Based on this definition, the distribution of cell cycle count number for the neuronal progenies of induced RGPs is given

$$P_n = A \sum_{g=0}^{\min(n_s,n)} 2^g F_g \sum_{m=1}^{\infty} Q_m \delta_{n,m+g} = A \sum_{g=0}^{\min(n_s,n)} 2^g F_g Q_{n-g}$$

where the normalization is given by $A^{-1} = \sum_{g=0}^{n_s} 2^g F_g$. Note that, here, the factor of 2^g accounts for the amplification of RGP number before entry into neurogene-

sis.

Predictions: Based on this analysis, what predictions can be made in relation to the iCOUNT data? In this context, there are two experiments to consider: In the first, cells are labelled with a ubiquitous promoter at E14.5 and then traced for around one day, 1.5 days and 2 days. In the second case, cells are labelled at E11.5 and then traced until E19.5. Based on the analysis of Gao et al., by E14.5, the vast majority if not all RGPs have already entered into their neurogenic phase while, at E11.5, many RGPs are still positioned within the amplifcation phase.

Starting with the E14.5 induction, if we focus on the 2-day chase, the majority of induced RGPs and all intermediate progenitors should have fully completed their neurogenic programme. Moreover, if we focus on labelled cells in the VZ/SVZ, we can expect that the majority will have derived from RGPs. In this case, the distribution of cell cycle number can be compared directly with the predicted probability distribution Q_n of induced RGPs. Taking $q = 0.3$ and $p = 0.2$, values that match the observed neuronal outputs of asymmetric MADM clones, we obtain a prediction that fits remarkably well with the observed distributions (Figure 4C).

Based on the MADM tracings, for the E11.5 induction, it is expected that some 50% of RGPs are expected to be in their amplification phase (with $a(t) \simeq 0$), while the remaining RGPs have already progressed to the neurogenic phase. With tissue fixed at E19.5, the vast majority of these RGPs should have completed their neurogenic program. Then, using the same estimates for the probabilities p and q, the predicted cell cycle number distribution is shown in Figure 4G alongside the estimated values from the iCOUNT system. Here, there is generally good

agreement between the model and the data, with the principal peak positioned around 3-4 rounds of division. However, the model predicts an excess of weight at higher numbers of cell divisions, consistent with the marking of cells deeper within the amplification phase.

This exposes a challenge for experiment since cells that have undergone multiple rounds of division would express very low levels of mCherry expression, where small fluctuations of intensity would alter dramatically the predicted cell cycle number. Nevertheless, the general quantitative agreement between the model pre-dictions (derived from the clonal data) and the experimental results obtained by the iCOUNT system emphasizes the potential of the latter to serve as a useful quantitative assay.
